# Spatio-temporal variation in the root-associated microbiota of orchard-grown apple trees

**DOI:** 10.1186/s40793-022-00427-z

**Published:** 2022-06-17

**Authors:** Maximilian Fernando Becker, Manfred Hellmann, Claudia Knief

**Affiliations:** 1 Institute of Crop Science and Resource Conservation - Molecular Biology of the Rhizosphere, University of Bonn, Nussallee 13, 53115 Bonn, Germany; 2 Dienstleistungszentrum Ländlicher Raum (DLR) Rheinpfalz, Kompetenzzentrum Gartenbau Klein-Altendorf, 53359 Rheinbach, Germany

**Keywords:** Rhizosphere, Endosphere, Spatio-temporal variation, Microbiota, Apple, Trees

## Abstract

**Background:**

The root-associated microbiome has been of keen research interest especially in the last decade due to the large potential for increasing overall plant performance in agricultural systems. Studies about spatio-temporal variation of the root-associated microbiome focused so far primarily on community-compositional changes of annual plants, while little is known about their perennial counterparts. The aim of this work was to get deep insight into the spatial patterns and temporal dynamics of the root associated microbiota of apple trees.

**Results:**

The bacterial community structure in rhizospheric soil and endospheric root material from orchard-grown apple trees was characterized based on 16S rRNA gene amplicon sequencing. At the small scale, the rhizosphere and endosphere bacterial communities shifted gradually with increasing root size diameter (PERMANOVA R^2^-values up to 0.359). At the larger scale, bulk soil heterogeneity introduced variation between tree individuals, especially in the rhizosphere microbiota, while the presence of a root pathogen was contributing to tree-to-tree variation in the endosphere microbiota. Moreover, the communities of both compartments underwent seasonal changes and displayed year-to-year variation (PERMANOVA R^2^-values of 0.454 and 0.371, respectively).

**Conclusions:**

The apple tree root-associated microbiota can be spatially heterogeneous at field scale due to soil heterogeneities, which particularly influence the microbiota in the rhizosphere soil, resulting in tree-to-tree variation. The presence of pathogens can contribute to this variation, though primarily in the endosphere microbiota. Smaller-scale spatial heterogeneity is observed in the rhizosphere and endosphere microbiota related to root diameter, likely influenced by root traits and processes such as rhizodeposition. The microbiota is also subject to temporal variation, including seasonal effects and annual variation. As a consequence, responses of the tree root microbiota to further environmental cues should be considered in the context of this spatio-temporal variation.

**Supplementary Information:**

The online version contains supplementary material available at 10.1186/s40793-022-00427-z.

## Introduction

The rhizosphere is defined as the narrow region of soil around plant roots in which the roots, the biota and the soil interact with each other [1]. It harbours a specific microbiome, which influences plant growth and development and has potential to contribute to sustainable agriculture [2–4]. Plants enrich microbial taxa from the surrounding soil, which then thrive in the root-associated soil and eventually establish a closer relationship by entering the root to pursue an endophytic lifestyle [5, 6]. This results in compartment-specific microbial communities with decreased microbial diversity and an expected higher level of interaction of root endophytes compared to the rhizosphere microbiome [5, 7]. The assembly of the plant root-associated microbiome depends on several deterministic factors such as plant host genotype and developmental stage, soil properties, plant cultivation practices, geographical location, possible presence of root pathogens and stochastic factors [8]. The impact of these factors on the root-associated microbiome has been studied in different herbaceous and annual plants [5, 9–13], but less in perennials and in particular in tree species [14–18].

In herbaceous and annual plants, temporal dynamics in the structure of the associated microbial community are considered to be closely linked to the plant development stage [8, 13, 19–22]. Previous studies on temporal dynamics of the root associated microbiota in trees have either focussed on the early assembly or on pathogen infection [23–25], and it remains unclear to what extent the root associated microbiota of trees is subject to seasonal changes and annual (year-to-year) variation.

Temporal dynamics in the root-associated microbiota of trees may result from seasonal shifts in carbon allocation into the roots and the surrounding soil [26]. This release of predominantly photosynthetic assimilates into the rhizosphere soil mainly occurs at root tips and in the elongation zone [26–28]. Older root sections become suberized and are considered to be less relevant compared to fine roots concerning carbon release into the rhizosphere and nutrient uptake. As rhizodeposition provides a major source of nutrients for microorganisms in the rhizosphere [28–30], the assembly of specific microbial communities likely varies between different root sections along the root axis. Such differences have been reported for some herbaceous monocots, e.g. along the root axis of maize [31] or between root tips and bases of *Brachypodium* [32], but remain to be assessed in the tree rhizosphere microbiota.

The root-associated microbiota of trees has so far predominantly been studied in poplar and orchard trees, especially in citrus and apple [7, 15, 17, 18, 23]. Most studies with apple focussed on apple replant disease, a worldwide phenomenon causing growth reductions and losses in fruit yield and quality [33–37]. The poplar studies mainly addressed the variability of bacterial communities between tree individuals and between plant compartments such as root, rhizosphere, leaf or stem [7, 15]. For a detailed study of the spatio-temporal variation in the tree-root associated microbiota, we chose mature apple trees (*Malus* X *domestica* Borkh.) as model organism, because of its high importance as perennial fruit crop with a worldwide production of 86 million tonnes in 2018 [38]. Commercially grown apple trees have a particular root architecture with very compact root growth (Additional file 1: Fig. S1), which facilitates a systematic spatial analysis of all parts of the root system.

Aim of this study was to systematically investigate spatio-temporal patterns in the root-associated microbiota of commercially grown apple trees. We hypothesized that (i) small-scale differences exist in the structure of the microbiota within individual tree root systems, with a successional gradient in relation to root diameter, as rhizodeposition is considered to decrease with increasing root size, (ii) the root-associated microbiota undergoes temporal succession, related to the phenological development of the plant, and shows annual variation, (iii) spatial patterns and temporal dynamics differ between the rhizosphere and endosphere microbiota, resulting from specific impacts of different factors on these microbiotas. To test these hypotheses, we analysed the root-associated microbiota of orchard-grown apple trees based on three field trials. Focus of the first trial (referred to as “spatial trial”) was the spatial variation within the root system of individual trees, while the second “temporal trial” addressed variation over time. In a third “spatio-temporal trial”, performed a year later, the small-scale spatial variation within the root system and the temporal patterns were further elucidated and directly compared. Moreover, larger-scale spatial variation from tree to tree resulting from field heterogeneity was assessed for trees between and along two rows, i.e. along an 80-m longitudinal transect in the field. We studied the spatio-temporal patterns comparatively in two compartments according to the concept of Donn et al. [19] by analysis of the loosely associated root microbiota (L-compartment), which primarily represents microorganisms residing in the rhizosphere, and the tightly associated microbiota (T-compartment), which primarily represents endophytes and microorganisms being very tightly associated to the root surface. Focus was on the bacterial community composition, which was analysed by amplicon sequencing of the 16S rRNA gene.

## Material and methods

### Study site and root sampling

Three field trials were conducted at the research facility “DLR Rheinpfalz” in Meckenheim, Germany. For the spatial trial, the entire root systems of four healthy adjacently grown apple trees (in order from tree 1 to tree 4) of the variety “Welland” were dug out and divided into four quadrants around the stem. In each quadrant, roots were divided into four sections depending on their root diameter (1: ≤ 1 mm, 2: 1–2 mm, 3: 2–4 mm, 4: ≥ 4 mm root diameter) as a proxy for root age and rhizodeposition. Triplicate samples were taken from each size category within a quadrant. One bulk soil sample was taken for each quadrant of each tree nearby the respective tree root system. For the temporal trial, root systems of six healthy trees of the variety “Topaz” were sampled at twelve time points over the course of one year from May 2018 to April 2019 (Additional file 1: Table S1). Trees were located in two opposing rows and in each row three adjacent trees at approximately 1.5 m spacing were sampled, thus all standing in close proximity to each other. Root samples were collected at each timepoint from each tree using a custom-made metal corer (Ø = 4.5 cm), which was inserted approx. 50 cm into the soil at a distance of around 20–30 cm from the tree trunk. Care was taken to sample from a new position within the tree root system at each time point. Roots with a size between 1 to 6 mm were collected from the drill core, primarily from 30 to 40 cm soil depth. In the spatio-temporal trial, nine healthy apple trees of the variety “Topaz”, located in the same two rows as the trees of the temporal trial were sampled four times between March and end of August 2019 (Additional file 1: Table S1). The timepoints reflect phenological stages from initial emergence of leaves in spring until shortly before fruit harvest, during which we expected to find the strongest temporal dynamics in the community composition. Nine trees were analysed within two opposing tree rows, respectively, spanning a distance of roughly 80 m. The rows were divided into three equally large clusters and three trees were sampled in each cluster, i.e. every third tree was sampled in each cluster. Root samples were collected with a metal corer and separated into two different size fractions in this trial: fine roots (FR) with a diameter between 1 and 3 mm and thick roots (TR) with 3 to 6 mm diameter.

### Sample processing

All samples were collected in 50-ml falcon tubes, stored on ice and frozen at -80 °C within six hours of sampling. Upon thawing for further processing, loosely attached soil was shaken off and the roots cut into the different root size fractions. Loosely and tightly root-associated microorganisms were collected according to the protocol of Donn et al. [19]. For the L-compartment, around 45 ml of 0.2 mM sterile CaCl_2_ solution was added to the root samples in 50-ml falcon tubes and vortexed three times for 30 s to loosen adhering soil and microorganisms from the roots. After 10 min of sedimentation the root material was transferred into a fresh 15-ml falcon tube, which was immediately frozen in liquid nitrogen for analysis of the T-compartment. The suspension containing the microorganisms of the L-compartment was centrifuged at 4255 × g for 15 min at 5 °C in a swing-bucket rotor to pellet all microbial cells. The supernatant was discarded up to 15 ml, the pellet resuspended in this remaining liquid by vortexing, the suspension transferred to a 15-ml falcon tube and centrifuged again. The supernatant was decanted and the pellet immediately frozen in liquid nitrogen. Both, L- and T-samples were stored at − 80 °C until further processing. To improve comparability, the bulk soil samples were processed in a similar way as the root samples by adding 45 ml of 0.2 mM sterile CaCl_2_ solution to the soil, vortexing three times for 30 s, followed by 10 min of sedimentation and centrifugation at 4255 × g for 15 min at 5 °C. The supernatant was discarded and the sample frozen in liquid nitrogen. All samples were freeze dried using a Heto PowerDry PL6000 freeze dryer (Thermo Fisher Scientific, Waltham, MA) and vortexed afterwards to homogenize the sample material. The frozen root material of the T-compartment was ground using a Retsch Mixer Mill MM 400 (Haan, Germany) and Retsch 25-ml grinding jars with 15-mm steel balls for 2 min at 25 Hz.

### DNA extraction and 16S rRNA gene PCR

A detailed description of the DNA extraction and subsequent 16S rRNA gene targeted PCR is given in the supplement. In brief, DNA extractions were performed using the NucleoSpin® Soil DNA extraction kit (Macherey Nagel, Düren, Germany) and DNA concentrations were quantified using the QuantiFluor®dsDNA System (Promega Corporation, Fitchburg, WI). For bacterial community analysis, the 16S rRNA gene was amplified using an LNA PCR protocol to suppress the amplification of plant organelle derived 16S rRNA genes [39]. The bacterial genes were amplified using the modified primer set 63f-1492r, followed by a nested PCR using primer set 799f-1193r (V5–V7 region) to obtain PCR products of adequate length for sequencing. The forward primer in this nested PCR contained an 8-bp sample-specific barcode (Additional file 1: Table S2), similarly as used in Frindte et al. [40]. PCR products were pooled at equimolar concentrations and purified with the HighPrep™PCR Clean-up System kit (MagBio Genomics, Gaithersburg, MD). Library preparation and sequencing on a HiSeq system (Illumina, San Diego, CA) was performed by the Max Planck-Genome-centre Cologne and generated paired-end reads (2 × 250 bp).

### Sequence data analysis

The raw sequence reads were processed using a custom bash script with Cutadapt version 2.10 to demultiplex the samples [41]. Primer removal and further processing was done with QIIME2 version 2021.02 [42]. Denoising was performed using DADA2, likewise as forward and reverse read trimming and truncation after inspecting the quality profiles according to the developer’s recommendations [43]. Amplicon sequence variants (ASVs) of each trial were defined using a custom classify-sklearn plugin classifier against the SILVA 138 database, which was subsetted to the amplicon region and using the last common ancestor method [44–46]. For each trial, the classified reads were quality filtered separately by removing rare ASVs that appeared less than 20 times and in less than five samples within a trial. Likewise, samples with less than 10.000 reads were excluded. The total number of samples, the hierarchical structures of the trials, the read numbers and the number of samples remaining after quality filtering are displayed in Additional file 1: Table S3. After quality filtering a minimum of 28 samples remained in the spatial trial for each root section in each compartment. In the temporal trial, between three and six samples were available for each timepoint in each compartment and between four and nine samples in the spatio-temporal trial for each timepoint in each root section and compartment.

Statistical analyses were performed in the QIIME2 environment and in R version 4.0.2 [47] using the packages “phyloseq” [48], “Microbiome” [49] and “qiime2R” [50], while figures were generated using the “ggplot2” package [51]. All further statistical analyses were done separately for the two root compartments and the bulk soil by dividing the datasets. Alpha diversity was estimated by Shannon`s diversity index using a feature table rarefied to 10.000 reads per sample. Linear mixed-effects models were constructed to assess differences in alpha diversity using the “lmer” function of the lme4 package for the spatial and spatio-temporal trial, while the “lme” function of the nlme package was used for the temporal trial. For the spatial trial, the Shannon values of the pseudo-replicates were averaged. The variables root section and plant individual were used as fixed factors and their interaction was included, while the root quadrant was added as random effect. In the temporal trial, tree individual and season (season defined as shown in Additional file 1: Table S1) were used as fixed factors, the tree individual as random factor and the date of sampling was integrated to adjust the temporal autocorrelation using the “corAR1” constructor. In the spatio-temporal trial, the root sections and sampling timepoints were used as fixed factors and their interaction included, while the tree individual was added as random factor. Significance was determined using the “anova” function and pairwise comparisons were performed by estimated marginal means using the “emmeans” function of the emmeans package.

Differences in the bacterial community composition were determined based on the non-rarefied dataset using the q2-plugin “DEICODE”, a form of Aitchison Distance [52], and constrained analysis of principle coordinates (CAP) using “capscale” in the “vegan” package [53]. Statistical differences were calculated using “adonis”, a form of one-way permutational multivariate analysis of variance (PERMANOVA), followed by a pairwise PERMANOVA with Benjamini–Hochberg correction for multiple testing using the “pairwise.adonis” function, resulting in adjusted *p*-values (*p*_adj_). All explanatory variables were coded as categorical factors. In the spatial trial, the effects of tree individual and root section were evaluated and permutations constrained by the factor root quadrant due to the nested design. In the temporal trial, variation due to sampling timepoint and tree individual were assessed, whereby permutations were constrained by the factor tree individual due to the repeated sample collection from the same individuals over time. This was also done in the spatio-temporal trial, where the effects of timepoint, root section and tree individual were analysed. To assess whether gradual changes between or along tree rows contribute to tree-to-tree variation the factor tree was replaced by row or longitudinal position and the results were compared. Pairwise differential abundance analysis at ASV level was performed for all trials using ANCOM-BC with detection for structural zeros turned on [54]. Conservative variance estimates of the test statistic were used and *p*-values were adjusted using Holm’s correction. While ASVs with a mean abundance of ≥ 0.1% in either the L- or T-compartment were included in the analysis of the spatial trial, a threshold of ≥ 0.3% mean relative abundance was applied in the analysis of the second and third trial, because of the slightly lower sample number and thus an increased false discovery rate (FDR) for low abundant ASVs. Similarly, differential abundance analysis was performed at family and phylum level comparing the L- and T-compartment in the different trials.

## Results

### Differences in the root microbiota between compartments at phylum and family level

**Fig. 1:**
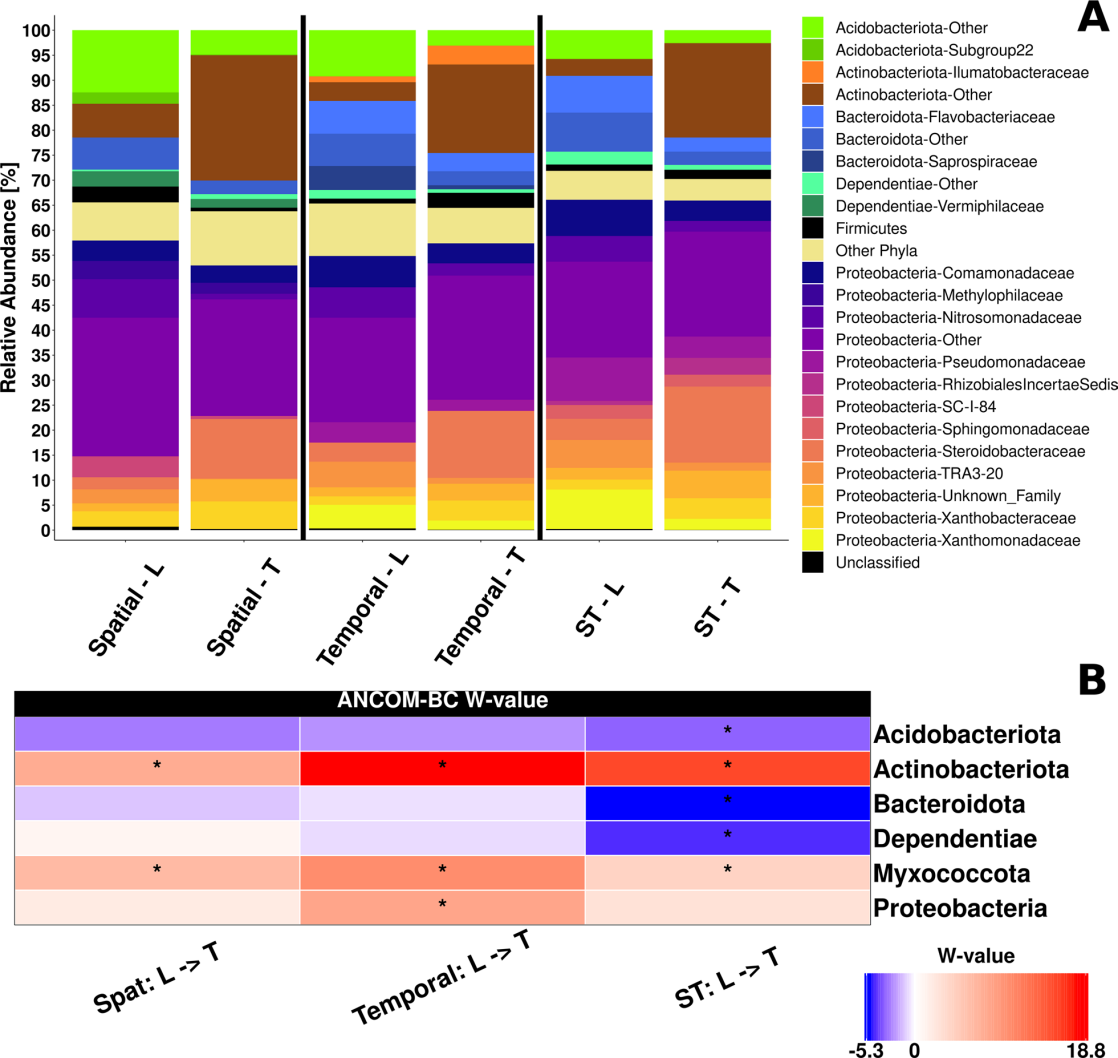
Root-associated bacterial community composition of apple trees as revealed by 16S rRNA gene amplicon sequencing. **A** Relative abundance of bacterial families in samples from three different field trials (Spatial, Temporal and ST: spatio-temporal) in the loosely associated (L) and tightly associated (T) compartment. Phyla and their families with < 2% relative abundance in the respective trial were grouped as “Other”. **B** Differential abundance analysis of L- and T-communities at phylum level using ANCOM-BC. The heatmap shows the coefficients obtained from the ANCOM-BC log-linear model divided by their standard error (called W-value). The colour code indicates differential abundances between two compartments with red indicating enrichment in the T-compartment. A “*” is shown if ANCOM-BC showed significant differences using the adjusted *p*-value in this comparison

Bacterial community composition was clearly dominated by members of the phylum *Proteobacteria* (mean relative abundance of 59.0%) in all samples, mostly followed by *Actinobacteriota* (12.7%), *Bacteroidota* (9.5%) and *Acidobacteriota* (6.9%) (Fig. 1A). Clear differences were seen in the community composition between L- and T-compartment in all three trials by one-way PERMANOVA on DEICODE distances (spatial trial: R^2^ = 0.483, *p* = 0.001 | temporal trial: R^2^ = 0.449, *p* = 0.001 | spatio-temporal trial: R^2^ = 0.458, *p* = 0.001). Most consistent was a strong increase in the relative abundance of *Actinobacteriota* and the low-abundant *Myxococcota* in the T-compartment according to a differential abundance analysis by ANCOM-BC (Fig. 1B). The corresponding analysis at family level revealed that 13 members of *Actinobacteriota* were enriched in the T-compartment in one or more trials. Phyla with significant enrichment in the L-compartment included the *Acidobacteriota* (Fig. 1B) with eight responsive families in at least one trial (Additional file 1: Fig. S2). Similar patterns were observed for the phyla *Bacteroidota* and *Dependentiae*. Moreover, individual families within the *Desulfobacterota*, *Gemmatimonadota, Firmicutes* and *Nitrospirota* responded in this way. In contrast, the proteobacterial families showed differential responses with 13 families being enriched and 14 being depleted in the T-compartment, which explains the mostly non-significant change at phylum level (Fig. 1B). Differences between the two compartments were also evident among the ten most abundant genera in each compartment (Additional file 1: Table S4). Due to these profound differences, which were also evident in CAP plots of individual trials (exemplarily shown in Additional file 1: Fig. S3), all further analyses were conducted separately for each compartment.

### Analysis of spatial patterns in the root-associated microbiota

**Fig. 2:**
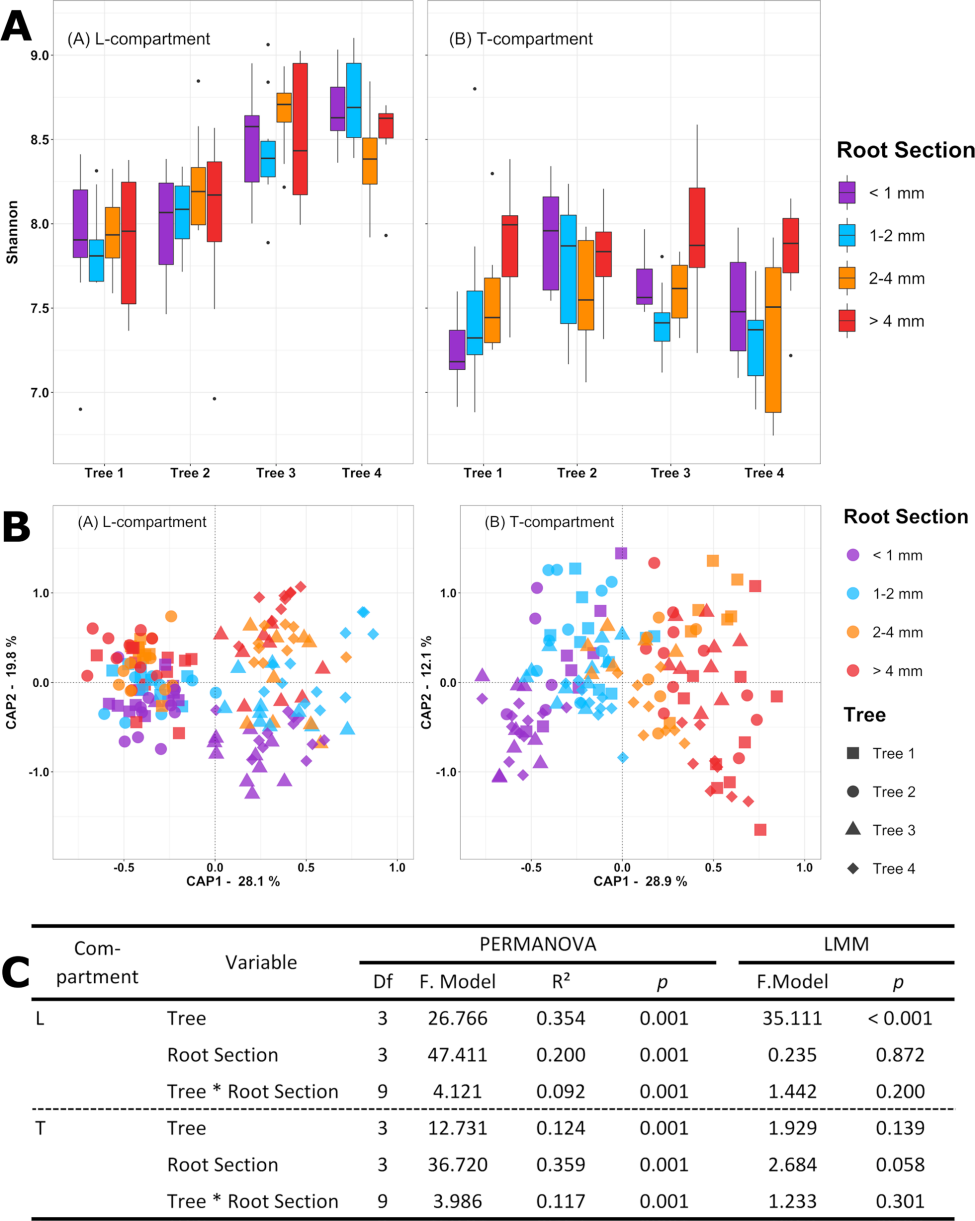
Spatial variation in the root-associated bacterial community of apple trees linked to root section, tree individual and root quadrant. **A** Variation in alpha diversity presented based on the Shannon index in the L-compartment (left) and T-compartment (right) of four different trees. The different colours in the boxplots indicate different root sections according to their root diameter. **B** Variation in beta diversity presented based on constrained analysis of principle coordinates (CAP; using DEICODE distance matrices, constrained by the variables tree, root section and root quadrant) shown for the L-compartment (left) and T-compartment (right). Different colours were used for different root size sections and symbol shapes for the four individual trees sampled. **C** Statistical evaluation of differences in alpha and beta diversity in the L- and T-compartment. Effect sizes in beta diversity were assessed by PERMANOVA based on DEICODE distance matrices, while differences in Shannon diversity were analysed based on Linear Mixed-Effects Models (LMM)

Spatial variation in the bacterial community structure within tree-root systems was assessed based on four adjacently grown apple trees. The root system of each tree was divided into four quadrants around the trunk and roots were separated into four size sections according to diameter. The mean Shannon diversity index of the bacterial community was significantly (*p* < 0.001) reduced in the T-compartment (7.61 ± 0.39) compared to the L-compartment (8.28 ± 0.43), where it was similar to the bulk soil diversity (8.22 ± 0.25). In the L-compartment, variation in diversity was observed between the individual trees (*p* < 0.001), while neither root section nor its interaction with individual trees caused significant changes (Fig. 2A, C). Pairwise comparisons showed that trees 1 and 2 had a slightly lower diversity estimate than trees 3 and 4 (*p* < 0.001). In the T-compartment, no factor showed effects on alpha diversity (*p*-values > 0.05) (Fig. 2C). Only the factor root section was close to the significance threshold (*p* = 0.058), with roots with the largest diameter tending to have a more diverse bacterial community than those of the other three size sections (Fig. 2A).

Analysing beta diversity by PERMANOVA and CAP (Fig. 2B, C) showed that tree-to-tree variation was the predominant factor explaining variation in bacterial community composition in the L-compartment (R^2^ = 0.354; *p* < 0.001), reflected by its separation along the first CAP axis, which explained 28% of the variation. Similar to alpha diversity results, the adjacent trees 1 and 2 as well as the adjacent trees 3 and 4 were more similar in their bacterial community composition to each other compared to the other two trees. This is supported by pairwise PERMANOVA, where all trees except tree 1 versus tree 2 were shown to be significantly different from each other (R^2^ values between 0.317 and 0.365, *p*_adj_ = 0.006) and with tree 3 versus tree 4 having a neglectable small R^2^ value (0.075, *p*_adj_ = 0.006) (Additional file 1: Table S5). Besides tree individuality, the root section had a significant impact on the bacterial community in the L-compartment (R^2^ = 0.200; *p* = 0.001), resulting in a successive separation of samples in CAP according to root size along the second axis with 19.8% explained variation. This gradual shift was most clearly seen in trees 3 and 4, which showed in general larger variation in community composition compared to trees 1 and 2 (Fig. 2B). Pairwise PERMANOVA performed over all four trees supported this, with strongest differences between the smallest and the two largest root sections (Additional file 1: Table S5). In the T-compartment, root section was the strongest explanatory factor (R^2^ = 0.359; *p* = 0.001), and a gradual shift in community composition in relation to root size section was evident in the CAP plot along the first axis, explaining 29% of the variation (Fig. 2B). Pairwise PERMANOVA showed that all root sections were indeed significantly different from each other (R^2^ values between 0.096 and 0.318; *p*_*adj*_ ≤ 0.012, Additional file 1: Table S5).

**Fig. 3:**
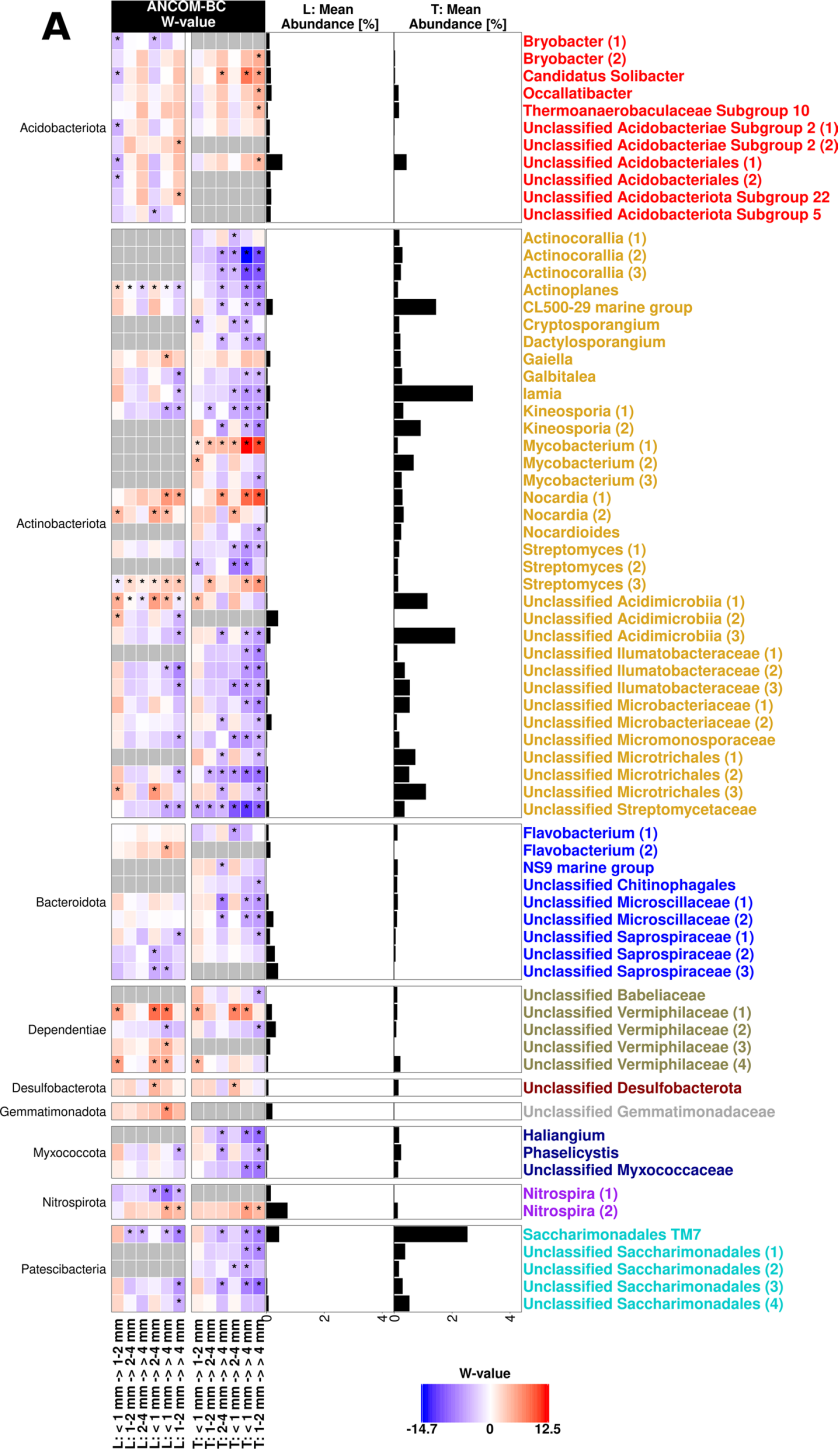

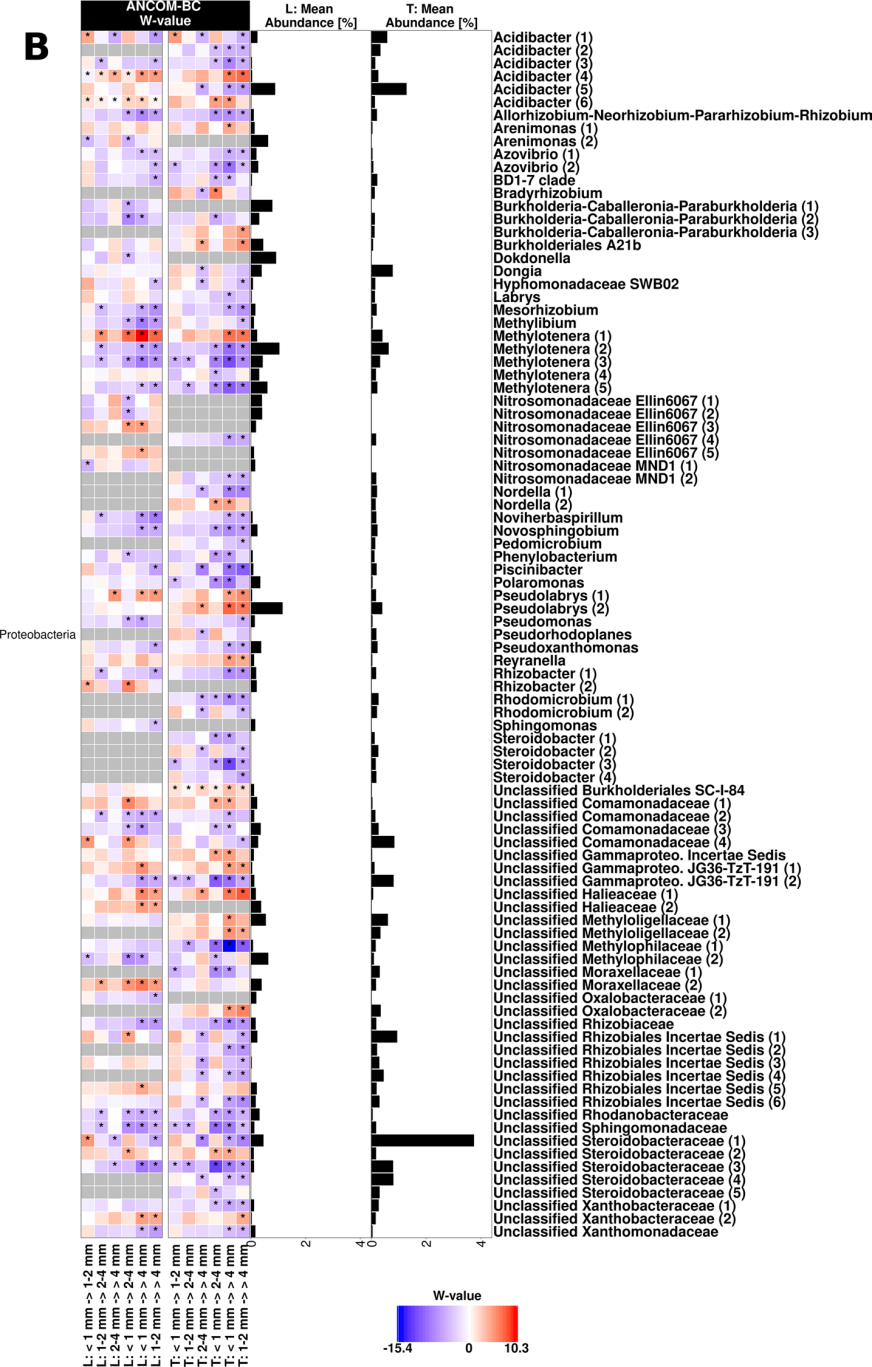
Differentially abundant ASVs in different root size sections of the L- and T-compartment based on ANCOM-BC. The heatmap shows the coefficients obtained from the ANCOM-BC log-linear model divided by their standard error (called W-value). A “*” is shown if ANCOM-BC showed significant differences using *p*_adj_-values in this comparison. The colour code indicates differential abundances between two root size sections with red indicating enrichment in the respective larger root section. A grey colour indicates that this ASV was not detected in the respective compartment. The mean relative abundance of the ASVs in the entire compartment is shown as % and ASVs with mean abundances ≥ 0.1% in either compartment are displayed. Names of ASVs are coloured according to phylum. **A** All phyla but *Proteobacteria*, which are displayed in (**B**)

ASVs with differential abundance between root size sections were identified based on ANCOM-BC. Interestingly, most ASVs with consistent changes across root size sections in the L-compartment showed a similar trend in the T-compartment (Fig. 3). Overall, the majority of responsive ASVs decreased in relative abundance with increasing root diameter in both compartments, e.g., *Actinobacteriota*, *Bacteroidota*, *Myxococcota* and *Patescibacteria*. In contrast, no phylum showed consistent increases with root size, rather some specific ASVs. In particular ASVs of the *Proteobacteria*, which was the most responsive phylum, showed differential responses. Most of the significant differences were observed between the largest and the two smallest root sections. In the L-compartment, this was seen for ASVs representing potential nitrogen fixing taxa (*Azovibrio*, *Mesorhizobium, Noviherbaspirillum*), methylotrophs (*Methylibium, Methylotenera,* unclassified *Methylophilaceae*), and other unclassified ASVs (e.g. *Rhizobiaceae, Sphingomonadaceae, Xanthomonadaceae*), which decreased in relative abundance in the largest root size fraction. ASVs that were in contrast more abundant in the largest size fraction included primarily *Acidobacteriota*, *Nocardia* and some *Proteobacteria* (*Acidibacter*, *Pseudolabrys*, one of the ASVs assigned to *Methylotenera* and unclassified *Halieaceae* and *Moraxellaceae*). In the T-compartment, the distinction of the largest root size fraction was even more prominent than in the L-compartment in all major responsive groups. Especially among the *Actinobacteriota* in the T-compartment, most ASVs decreased along the root section gradient towards the largest one with only *Nocardia, Mycobacterium* and one *Streptomyces* ASV being an exception, which increased along the size gradient. Furthermore, different *Proteobacteria* such as rhizobia, some methylotrophs, sphingomonads, or ammonium oxidizers tended to decrease in relative abundance in the larger root sections, while only few *Proteobacteria* increased (*Pseudolabrys, Reyranella*, unclassified *Halieceae* and *Methyloligellaceae*).

Seeing substantial tree-to-tree variation especially in the L-compartment, we assessed whether the bulk soil samples collected within the root quadrants showed a similar pattern. PERMANOVA and CAP (Additional file 1: Fig. S4) revealed indeed a grouping of the bulk soil samples according to tree location (R^2^ = 0.515; *p* < 0.05). Similar as seen in the L-compartment, samples taken nearby trees 1 and 2 tended to cluster more closely together compared to trees 3 and 4. Despite a rather low number of bulk soil samples being available, differential abundance analysis allowed us to identify 26 responsive ASVs with a mean relative abundance of ≥ 0.1% (Additional file 1: Fig. S5). Of these, 42.4% showed a similar response pattern in the L-compartment, while only 11.5% responded similarly in the T-compartment. Thus, tree-to-tree variation, especially in the L-compartment, appears to be influenced by spatial patterns in the bulk soil.

### Succession of the microbial community composition over time

**Fig. 4:**
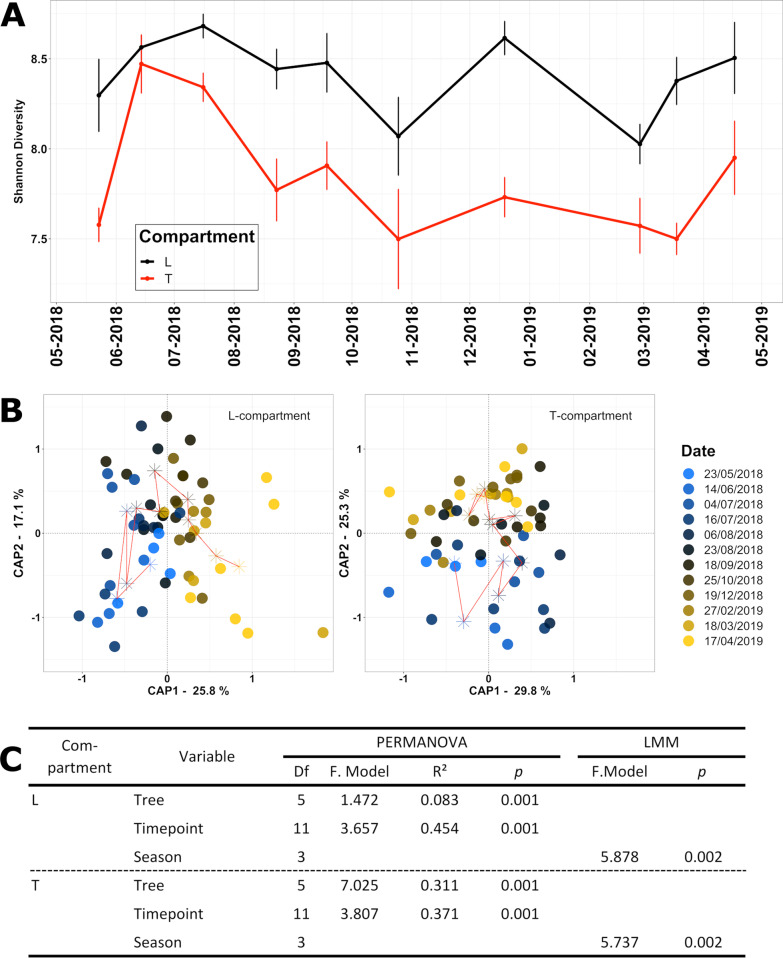
Temporal variation in the root-associated bacterial community of apple trees. **A** Changes in alpha diversity based on the Shannon index in the two root compartments over time. Error bars indicate the standard error. **B** Constrained analysis of principle coordinates (based on DEICODE distance matrices and constrained by the variables tree and timepoint) to assess the relevance of time on variation in bacterial community composition in the L-compartment (left) and T-compartment (right). A colour gradient differentiates the twelve sampling timepoints. The stars are the calculated centroids of the samples from each timepoint and are connected with a red line along the timeline. **C** Statistical evaluation of differences in bacterial alpha and beta diversity in the L- and T-compartment. Effect sizes in beta diversity were assessed by PERMANOVA based on DEICODE distance matrices, while differences in Shannon diversity were analysed based on linear mixed models

The temporal dynamics in the loosely and tightly root-associated bacterial communities were studied based on six apple trees that were repeatedly sampled twelve times over the course of one year (Additional file 1: Table S1). The alpha diversity estimates of both compartments showed comparable fluctuations over time with highest Shannon indices in the summer months June until August (Fig. 4A). Afterwards, diversity decreased with the exception of the December samples until around March/April, when a steep increase followed in spring around the time when leaves and first buds emerged. The statistical analysis showed significant results between seasons in both compartments (both *p*-values = 0.002) with the winter season having a significantly reduced diversity compared to the summer season in both compartments (both *p*-values = 0.001), even though the December timepoint showed an intermediate increase. Regarding beta diversity, the factor time showed a significant impact on the bacterial communities in both compartments according to PERMANOVA (L: R^2^ = 0.454; *p* = 0.001 | T: R^2^ = 0.371; *p* = 0.001). CAP plots revealed that fluctuations in the L-compartment occurred primarily in summer 2018, followed by a continuous though weaker succession in the following months until the end of the experiment in spring 2019 (Fig. 4B). In the T-compartment successional changes were likewise mostly seen between May and September 2018, while less changes occurred in the cold months. In PERMANOVA no pairwise comparison remained significant after adjusting for multiple testing considering all sample combinations. However, comparisons based on non-adjusted *p*-values revealed some trends, i.e. that changes developed primarily between summer (23.05.2018 to 16.7.2018) and autumn/winter timepoints (25.10.2018 to 17.4.2019) (Additional file 1: Fig. S6). In particular for the L-compartment, the bacterial community composition did not (yet) return to its initial pattern after one year, as seen in the CAP plot (Fig. 4B) and the non-adjusted *p-*values of the PERMANOVA, where the last timepoint (17.04.2019) was different from nearly all earlier timepoints.

**Fig. 5:**
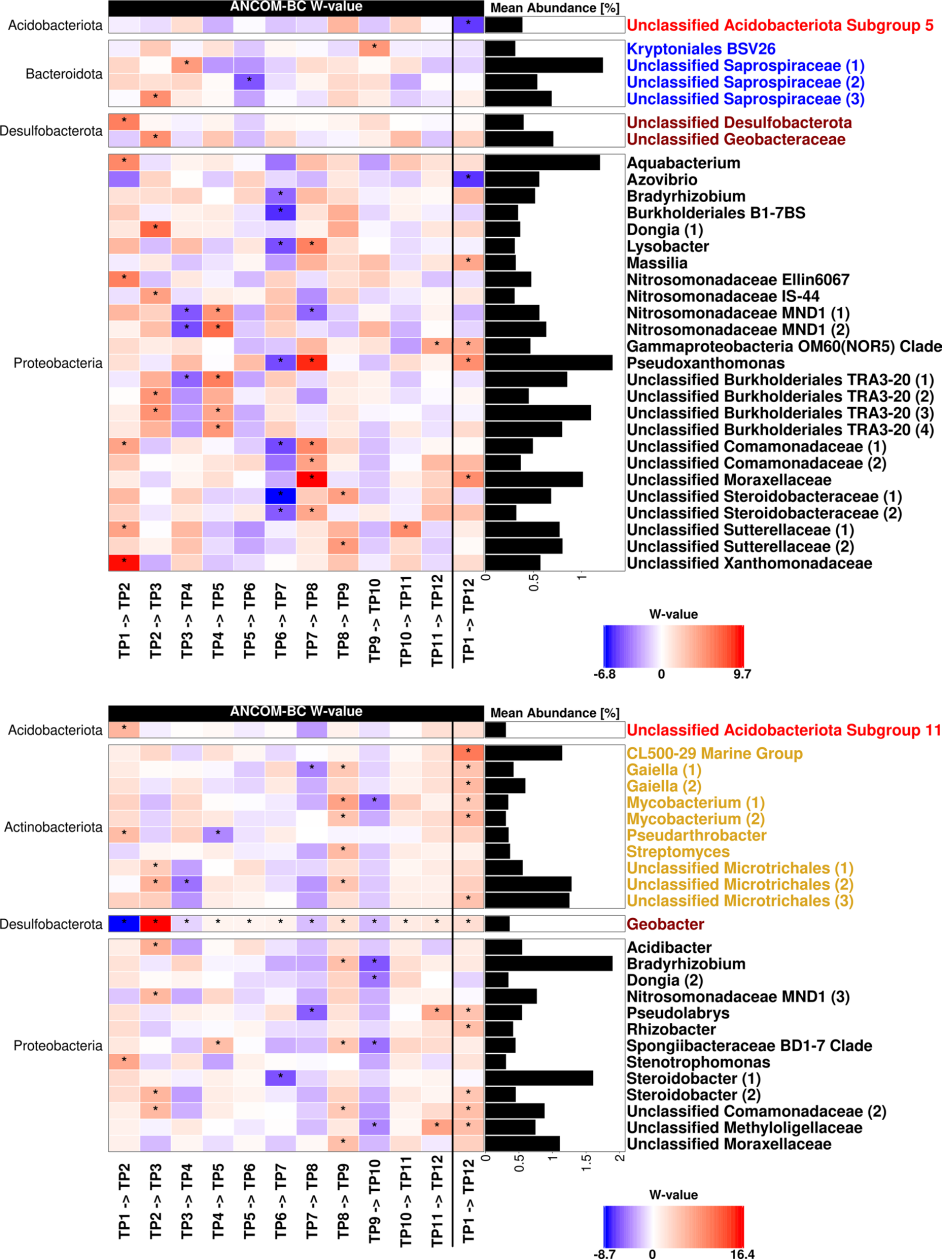
Differentially abundant ASVs between twelve successive timepoints in the L-compartment (upper) and T-compartment (lower panel) according to ANCOM-BC. The dates of the timepoints (TP) are listed in Additional file 1: Table S1. The heatmap shows the coefficients obtained from the ANCOM-BC log-linear model divided by their standard error (called W-value). A “*” is shown if ANCOM-BC showed significant differences using the *p*_adj_-value. The colour code indicates differential abundances between two samples with red indicating enrichment at the later timepoint. The mean relative abundance of the ASVs in the entire compartment is shown as % and ASVs with mean abundances ≥ 0.3% are displayed. The ASVs in the rows of the heatmap are separated according to phylum. Besides the comparisons between successive timepoints, differences between the first and last timepoint are shown

Temporal dynamics of abundant ASVs (mean relative abundance ≥ 0.3%) between successive timepoints were evaluated by ANCOM-BC (Fig. 5). Changes were primarily evident for *Proteobacteria*, while *Actinobacteriota* responded primarily in the T-compartment, where they occurred more abundantly (Fig. 1). In both compartments, we observed several taxa with recurrent fluctuations in relative abundance over one season, whereby a significant increase was quite often immediately followed by a significant decrease or vice versa (e.g. *Burkholderiales* TR3-20, *Nitrosomadaceae* MND1, *Lysobacter* or *Pseudoxanthomonas*). These changes correspond to the fluctuations observed in the CAP plot during summer (Fig. 4B, left panel). In the L-compartment, less significant changes were detected in autumn/winter (25.10.2018 to 17.04.2019; TP8 to TP12) than in spring/summer (Fig. 5, upper panel). The responsive taxa and their patterns in the T-compartment were mostly different from those in the L-compartment and most changes were seen between the first and last sampling timepoint (TP1 and TP12), where twelve significantly differentially abundant ASVs were identified.

Tree-to-tree variation in beta diversity was mainly seen in the T-compartment (R^2^ = 0.311; *p* = 0.001) (Fig. 4C). Pairwise PERMANOVA as well as ANOM-BC revealed that mainly tree individual 2 and partially tree 1 were distinct from the rest (Additional file 1: Fig. S7B). Interestingly, the most responsive abundant ASV (mean relative abundance in the T-compartment 2.2%, SD: 5.1) belonged to the genus “*Candidatus* Phytoplasma”, a plant pathogen. It was significantly enriched in tree 2 and to lower degree in tree 1 compared to all other trees (Additional file 1: Fig. S7B) reaching 24.4% relative abundance in tree 2 at the final sampling timepoint.

### Comparative analysis of temporal and differently scaled spatial variation in the rhizosphere microbiota

**Fig. 6:**
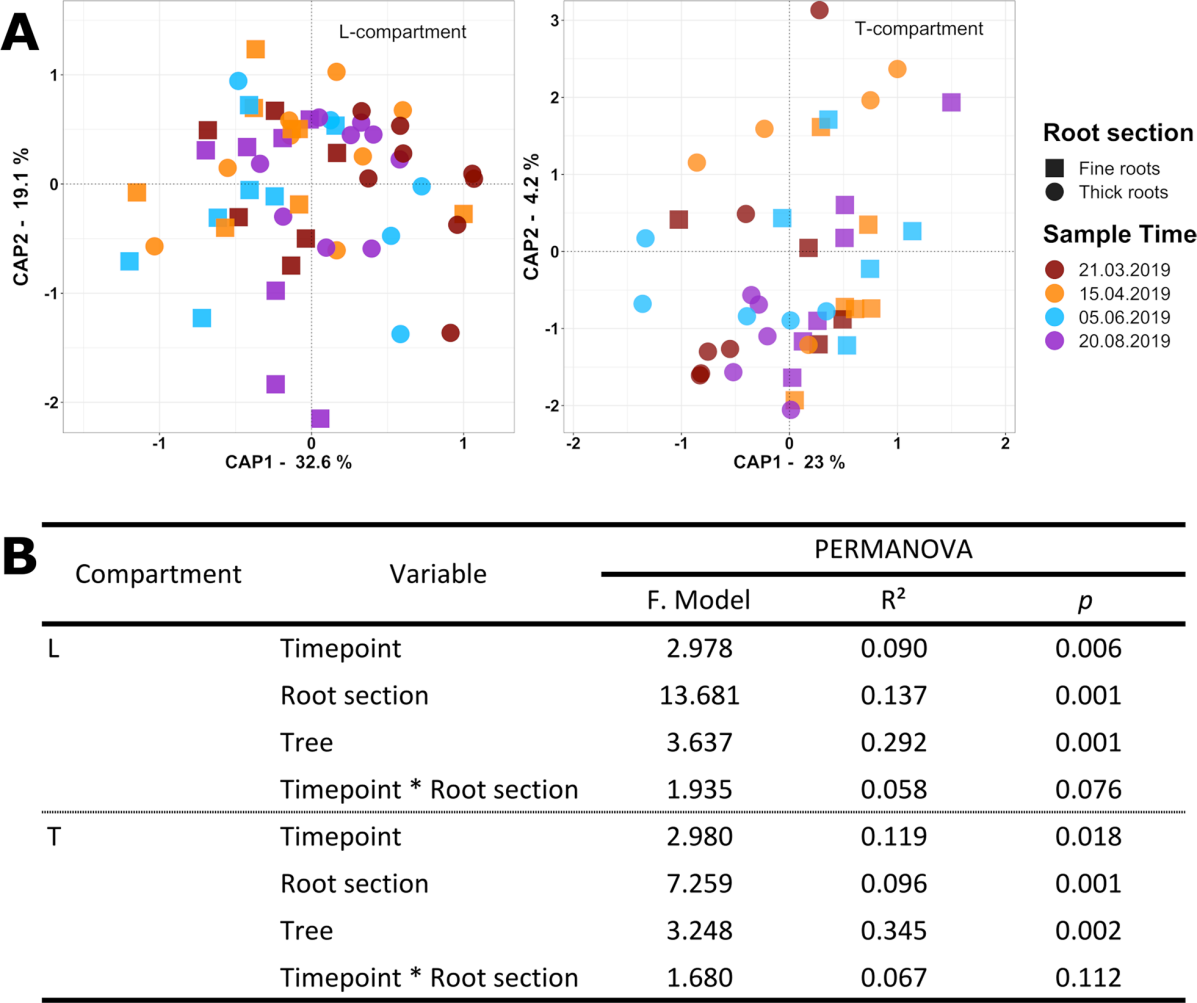
Variation in the apple root-associated bacterial community structure due to spatial (root section), temporal and tree-to-tree effects. **A** Constrained analysis of principle coordinates (CAP, based on DEICODE distance matrices, constrained by the variables tree, root section, and timepoint) shown for the L-compartment (left) and T-compartment (right). A colour gradient differentiates the four sampling timepoints and symbol shapes the root section. **B** Statistical evaluation of differences in bacterial beta diversity in the L- and T-compartment. Effect sizes were assessed by PERMANOVA based on DEICODE distance matrices

In the spatio-temporal trial we focussed on four time points in spring and summer, i. e. during the growing season, and on two relevant root size classes to assess small-scale spatial variation. In addition, larger-scale tree-to-tree variation was assessed along a longitudinal transect following two adjacent tree rows in the orchard (Additional file 1: Fig. S1) and evaluated between individual trees, trees between rows and along rows. Alpha-diversity of the bacterial communities was not changed by any factor in this trial, likewise as in the temporal trial. Beta diversity analysis based on a PERMANOVA model with root section, timepoint and tree individual as explanatory variables revealed that all three factors were significant in both compartments with tree-to-tree variation being most relevant (L: R^2^ = 0.292; *p* = 0.001 | T: R^2^ = 0.345; *p* = 0.002) (Fig. 6B). This was followed by root size in the L-compartment and time in the T-compartment. To evaluate whether tree-to-tree variation was following a spatial pattern between or along rows, these factors were used alternatively in the PERMANOVA model instead of tree. In the L-compartment, the position of a tree in the row as well as its interaction with the factors timepoint and root section was explaining a considerable part of the variation (Additional file 1: Table S6), while this was not evident in the T-compartment. To analyse variation comparatively in the two root size sections, the data were divided according to root section. PERMANOVA showed that time and tree individual explained variation only in the thick root section of both, the L- and T-compartment, but not in the fine root fraction (Table 1).

**Table 1:** Significant differences in the apple root-associated bacterial community structure due to spatial (longitudinal position in field), temporal and tree-to-tree effects

Compartment-section	Variable	PERMANOVA		
		F. Model	R^2^	*p*
L-FR	Timepoint	1.880	0.148	0.101
	Tree	2.052	0.431	0.365
L-TR	**Timepoint**	**4.903**	**0.244**	**0.001**
	**Tree**	**3.441**	**0.457**	**0.001**
T-FR	Timepoint	1.385	0.127	0.625
	Tree	2.311	0.566	0.763
T-TR	**Timepoint**	**2.947**	**0.279**	**0.012**
	**Tree**	**1.610**	**0.406**	**0.005**

Differences in bacterial diversity in the L- and T-compartment of fine (FR) and thick (TR) roots were assessed. Effect sizes were analysed by PERMANOVA based on DEICODE distance matrices. Significant results are printed in bold

In ANCOM-BC, we evaluated differences of highly abundant ASVs (mean abundance ≥ 0.3%) in samples of different root diameter or time (Additional file 1: Fig. S8). Significant root-section related differences were primarily seen at the first timepoint, with similar trends being mostly maintained at all other timepoints. As in the spatial trial (Fig. 3), most responsive ASVs were detected among the *Proteobacteria*. These included in the L-compartment ASVs of *Methylotenera, Pseudoxanthomonas*, and *Xanthomonadaceae*, which decreased in relative abundance in the thicker roots, while *Pseudolabrys* increased. In the T-compartment, ASVs identified as *Methylotenera* and several members of *Steroidobacter* and *Steroidobacteraceae* decreased in the thicker root sections consistently in both trials. Lastly, temporal dynamics were assessed independently in the two root size sections (Additional file 1: Fig. S8), revealing that temporal dynamics were mostly seen in the thick root fraction of the L-compartment. Changes were predominantly detected among the *Proteobacteria*, as in the temporal trial (Fig. 5). This included ASVs such as *Dongia*, *Pseudoxanthomonas*, *Burkholderiales* TRA3-20, *Nitrosomonadaceae* MND1 and unclassified members of *Saprospiraceae*, *Moraxellaceae* and *Comamonadaceae*, which were all responsive in both trials, though at different timepoints.

## Discussion

### Compartment-specific differences in the apple tree rhizosphere

Based on knowledge from annual crops [19, 32], we expected clear differences between the loosely and tightly associated bacterial communities. Indeed, consistent differences in relative abundances were observed for the dominant families and phyla in all three experimental trials (Fig. 1 and Additional file 1: Fig. S2). This is largely in accordance with differences reported for annual crops including maize, rice, wheat or *Arabidopsis*, where *Proteobacteria* and *Actinobacteriota* were more prevalent in the T-compartment or endosphere, while *Acidobacteriota* were more prevalent in the L-compartment or rhizosphere [5, 11, 12, 19, 55]. Moreover, our findings are in agreement with results from citrus and olive trees [56, 57], while they are partially different to the findings reported for poplar trees, which showed a significantly higher relative abundance of *Actinobacteriota* in the rhizosphere soil compared to the root endosphere, while *Proteobacteria* responded similarly as in our work [7, 58]. Overall, it appears that trees enrich similar phyla in the endosphere as many of their annual herbaceous counterparts, pointing towards analogous selection mechanisms.

By far the most abundant taxon (11–16%) in the T-compartment in all three trials was the genus *Steroidobacter* (or *Steroidobacteraceae* in the spatial trial) (Additional file 1: Table S4). This genus has been reported to be associated with apple roots in an earlier study [59], but not as the dominant taxon, and has been found abundantly in *Marchantia* liverworts, indicating a possible long co-evolutionary history with plants [60]. Growth of *Steroidobacter agariperforans* can be stimulated in vitro by diffusible metabolites of *Rhizobiales* [61], another typical group of plant root colonizers that were abundantly present in the apple tree root endosphere (Additional file 1: Table S4). Moreover, the genus is known from *Pinus* roots growing in subsoil or from the subsoil itself, with positive effects on nutrient cycling [62, 63]. Most of our sampled root material was located in the subsoil, which may promote the dominance of *Steroidobacter* in the T-compartment. Several other dominant taxa in the T-compartment in all three trials (e.g. *Acidibacter*, *Flavobacterium*, *Pseudomonas, Bradyrhizobium*, *Bacillus,* as well as unidentified ASVs in the families *Burkholderiaceae, Microtrichales* or *Rhizobiales* incertae sedis) have frequently been found in other rhizosphere studies and many of those are known to have plant beneficial traits, e.g. stimulation of plant defence, or have been reported to reduce the abundance of soil-borne pathogens in apple orchards (e.g. *Alternaria mali*) and may thus play an important role in the apple root microbiome [64–67].

In contrast, only few of the dominant taxa in the L-compartment have potentially plant beneficial traits such as *Pseudomonas, Flavobacterium*, members of *Burkholderiales*, *Comamonadaceae* or *Saprospiraceae*. They have been shown to utilize root metabolites, degrade (lignin-derived) aromatic compounds, produce anti-microbial substances, partake in sulfonate cycling in the wheat rhizosphere or break down complex organic compounds [32, 67–72]. Other dominant ASVs found here were *Nitrospira* and *Nitrosomonadaceae* MND1, capable of nitrification [73–75] and possibly important for nutrient cycling in the rhizosphere.

To conclude, both compartments harbour specific microbial communities with possible abilities to benefit the plant. Predominant taxa in the L-compartment are primarily known to be associated with the conversion of plant-derived organic compounds, are involved in the nitrogen cycle or may have growth-promoting traits, while several taxa in the T-compartment have been shown to harbour strains being involved in biocontrol or possess plant-growth promoting abilities.

### Bacterial community shifts along a root size gradient

We observed successive changes in beta diversity with increasing root diameter in the first and third trial in both compartments (Figs. 2, 6). This indicates that apple trees selectively shape their bacterial communities along the root axis. Differences between root sections were recently also reported for the maize rhizosphere microbiota, though the community did not show a gradual transition in beta diversity along the axis [31]. However, in that work the focus was on very early assembly processes and the analysed root regions were younger. In the apple rhizosphere, several ASVs showed similar trends of increasing or decreasing abundance in both compartments along the root size gradient (Fig. 3), suggesting that similar selection processes contribute to these differences along the root axis in both compartments.

In the L-compartment, the selective process is likely primarily driven by rhizodeposition, including processes such as organic carbon exudation, which changes and decreases with increasing root diameter as larger roots become suberized [76–78]. This is supported by our finding that rhizosphere-associated taxa, which are known to be frequent colonizers and relative abundant at early growth stages in other plants or to profit from root exudates, decreased towards the largest root section, e.g. *Allorhizobium-Neorhizobium-Pararhizobium-Rhizobium*, *Pseudoxanthomonas*, *Methylibium* or other unclassified *Rhizobiaceae* [79–82].

That carbon source availability can drive differentiation along the root axis in both compartments can also be nicely exemplified by focusing on methylotrophic bacteria. Several methylotrophic taxa occurred with higher relative abundance in the smaller root size sections in the two trials, such as *Methylotenera*, *Methylibium* or members of the family *Methylophilaceae* (Fig. 3 and Additional file 1: Fig. S8). These organisms can grow on methanol, which is a waste metabolic by-product of growing plant tissue [83, 84]. Using methanol likely provides a selective advantage for methylotrophs during plant colonization, as demonstrated earlier [85]. Since root growth occurs predominantly at the root tips and in the elongation zone, this is likely the area with the highest release of methanol and corresponding very well to the enrichment of methylotrophs in thinner root sections. Noteworthy, not all ASVs representing methylotrophs were enriched towards these thinner root sections, few taxa showed an opposite pattern (ASVs identified as *Methylotenera* or *Methyloligellaceae*), which may indicate further niche differentiation within the population of methylotrophs. It is likely that other taxa respond to other carbon compounds in the L- and T-compartment in a similar way, but these specific dependencies, which may involve more than one carbon compound a taxon is responding to, remain to be identified in future studies.

### Temporal changes in the loosely and tightly bound root microbiota

The temporal trial, which focused on differences throughout an entire year, showed that a succession in community composition took place in both compartments with more changes occurring during spring and summer than in winter (Figs. 4, 5). This resulted in a higher alpha diversity during the summer months (Fig. 4A) and in community compositional differences primarily between summer and winter (Fig. S6). It is likely that the changes in the root-associated microbiota were related to plant phenological processes. Apple root growth in mature trees has been reported to occur unevenly during the year with a possible bimodal pattern with substantial root growth (“root flush”) around full bloom and either mid-summer or harvest [86]. This coincides with the increasing Shannon diversity observed during spring and early summer in 2018 and 2019 (Fig. 4A) [84]. Likewise, changes in community composition were stronger during the growing season, especially in the L-compartment, i.e. at the time when photosynthesis and rhizodeposition were most relevant [87].

Interestingly, the bacterial communities of the L-compartment at the last analysed timepoint shifted further away from most other timepoints rather than returning to its initial state (Fig. 4B, Additional file 1: Fig. S6), indicating that the loosely associated microbial community does not necessarily return to a highly season-specific state after one year. This is underlined by the fact that no taxon that was responsive to season in the temporal trial showed a highly reproducible pattern in the spatio-temporal trial, though the identity of the responsive taxa was at least partially overlapping in the two trials. Microorganisms in the L-compartment are known to be less protected against abiotic influences compared to the T-associated microbiota [88], which suggests that further factors besides tree phenology are inducing changes. Variations in weather conditions are likely contributing to these year-to-year alterations. We encountered quite unordinary weather conditions in 2018 with high temperatures during summer with little precipitation and a warm December with intensive precipitation [89]. The latter may have caused the spike in alpha-diversity in the L-compartment in December 2018 (Fig. 4A) and perhaps also the intermediately higher number of responsive taxa in the T-compartment (Fig. 5, T8 to T9 and T9 to T10). Besides a direct impact of weather conditions on microorganisms, higher temperatures increase the rate of photosynthesis and thus likely the rate of rhizodeposition, as shown for perennial plants and trees [90, 91]. An increased carbon flux due to higher temperatures is thus likely contributing to seasonal dynamics in bacterial communities. Water availability is also strongly weather related, but drought was probably not a major limiting factor in this study even during the exceptionally warm summer, because the trees were drip-irrigated. It may primarily have affected the dynamics in December 2018. Taken together, our results suggest that the temporal dynamics in the root-associated microbiota are related to plant phenology as well as abiotic factors such as weather conditions, which can act directly on the microbiota or indirectly via modulating plant physiological processes.

In the third trial, we additionally evaluated temporal differences in the root-associated microbiota individually for different root size fractions. Seasonal dynamics were detected in both compartments (L and T) (Fig. 6B), but largely restricted to the thicker root fraction (Table 1). Likewise, tree-to-tree variation was limited to the thicker root fraction. These findings indicate that tree-specific communities may develop with increasing root thickness/age, which are then more responsive to seasonal dynamics compared to the microbial communities in finer roots. That no significant differences were observed in the fine roots in either compartment indicates that other factors than seasonal dynamics influence the microbiota there.

### Field scale gradients and pathogen infection as underlying causes for tree-to-tree variation

In all three trials we observed a significant impact of the individual trees on the bacterial community structure (Figs. 2, 4, 6), with varying effect sizes on the L- versus T-compartment. In the spatial trial, tree-to-tree variation was primarily seen in the bacterial community of the L-compartment. This was likely caused by underlying variation in the bulk soil microbiota, because the microbiota in the bulk soil samples showed similar community patterns as in the L-compartment (Additional file 1: Fig. S4), and because the rhizosphere microbiota is known to be drafted from the surrounding bulk soil [92]. Similarly, the observed variation along rows in the spatio-temporal trial points to field-scale heterogeneity in the bulk soil that causes shifts especially in the loosely associated microbiota (Fig. 6B and Additional file 1: Table S6). A previous study analysing the spatial structuring of soil microbial communities in an apple orchard also found that spatial (1–5 m) variability was present within an orchard, though it did not follow a predictable pattern [65]. Such variability can be explained either by physio-chemical differences in the soil or by the fact that the distribution of soil microorganisms relies on passive mechanisms of dispersal in the soil, even in the absence of environmental gradients [93].

In all trials, tree-to-tree variation was observed. In the temporal and spatio-temporal trial, this variation was more relevant in the T-compartment than in the L-compartment (Figs. 4C, 6B). Moreover, it was of higher relevance in the thicker than in the thinner root size fractions (Table 1). Similar differences were also seen in the first trial (data not shown) and indicate that tree-specific influences may predominantly affect closely associated endophytes in older root regions. We identified pathogen infection as likely cause for tree-to-tree variation in the temporal trial, as two trees showed high relative abundance of an ASV classified as “*Candidatus* Phytoplasma”. This genus includes the apple specific root pathogen “*Candidatus* Phytoplasma mali”, the causal agent of apple proliferation and a BLAST search of the sequence resulted in 100% query cover [59, 94]. We did not detect any signs of infection when sampling, but infected trees may be asymptomatic. Tree-to-tree variation of the tightly associated bacterial community is likely caused by restructuring the native community in infected trees, as seen in previous studies [95–97], and it highlights the impact of a pathogen infection in the root system on the root-associated microbiome. This pathogen was not found or in marginally small abundances in the other two trials, which leads to the conclusion that further tree-specific traits cause tree-to-tree variation. Observing tree-to-tree variation in a comparable range as seen for the impact of root section and temporal variation indicates the need for a sufficiently high number of replicate trees in future orchard studies.

## Conclusion

Our study demonstrates that the root-associated bacterial microbiome of apple trees is compartment-specific and shows spatio-temporal patterns. Genera being associated with the conversion of organic carbon compounds or being involved in the nitrogen cycle were more frequently enriched in the L-compartment, while several genera in the T-compartment are known to include strains being involved in biocontrol or with plant-growth promoting abilities. Spatial patterns were shown to exist at different scales, ranging from variation within the root system of an individual tree over tree-to-tree variation between adjacent trees to variation relevant to field scale. We identified root diameter, which served as proxy for root age and therewith differences in root physiology and rhizodeposition processes, as a relevant factor for variation within the tree root system. Factors leading to tree-to-tree variation act compartment specific, with soil properties introducing spatial patterns primarily in the loosely associated rhizosphere microbiota, while tree-specific traits such as pathogen infection level introduced more variation in the tightly associated microbiota. Seasonal variation is also present in the microbiota of both compartments of apple trees, most evident between summer and winter, likely linked to tree phenology, weather conditions, and climatic differences between years. This temporal variation may modulate microbiome responses to other environmental factors and deserves careful attention in future field studies. Besides, the observed tree-to-tree variation, which was often as relevant as the spatio-temporal variation, points to the need for sufficiently large sample sizes even within one orchard. Variation in microbiome data due to root region or time can be reduced by collecting homogenous samples, e.g. with a consistent representation of root regions. Such strategies will help to identify further influence factors of the tree root microbiome in future studies.

## Supplementary Information

**Additional file 1. Figure S1.** Photographs showing the root system of a fully grown commercial apple tree (top) and two rows of an apple orchard (bottom). **Figure S2.** Differential abundance analysis of the loosely (L) and tightly (T) associated bacteria in the three experimental field trials using ANCOM-BC. The heatmap shows the coefficients obtained from the ANCOM-BC log-linear model divided by their standard error (called W-value) with red indicating enrichment in the T-compartment. A is shown if ANCOM-BC showed significant differences using the *p*_adj_-value in this comparison. The mean abundance of the families in their respective trial are shown in the adjacent barplot as % and only families with mean abundances ≥ 0.5% are shown (ST refers to the spatio-temporal trial). A greyed-out field means that this family is below the 0.5% threshold in a trial. The families in the heatmap rows are separated by the phylum they belong to and displayed in different colours. **Figure S3.** Root-associated bacterial community composition of the loosely (L) and tightly (T) associated bacteria in four different root size sections and the bulk soil (b) of four apple trees analysed in the spatial trial. Constrained analysis of principle coordinates (CAP; based on DEICODE distance matrix and the variables compartment, tree and root quadrant) to assess the relevance of those variables on variation in bacterial community composition. **Figure S4.** Root-associated bacterial community composition of bulk soil near the apple trees analysed in the spatial trial. Constrained analysis of principle coordinates (CAP; based on DEICODE distance matrix and the variables tree and root quadrant) to assess the relevance of those variables on variation in bacterial community composition. **Figure S5.** Differential abundance analysis of the loosely (L) and tightly (T) associated bacteria and the bulk soil (B) in four different trees (T1 to T4) of the spatial trial using ANCOM-BC. The heat map shows the coefficients obtained from the ANCOM-BC log-linear model divided by their standard error (called W-value). A “*” is shown if ANCOM-BC showed significant differences using the *p*_adj_-value in this comparison. The colour code indicates differential abundances between two samples with red indicating enrichment in the larger root sections. A grey colour indicates that this ASV was not detected in the respective compartment. The mean relative abundance of the ASVs in the entire compartment is shown as % and ASVs with mean abundances ≥ 0.1% in either compartment are displayed. The ASVs in the rows of the heat map are separated according to phylum. **Figure S6.** Pairwise PERMANOVA for comparison of time points in the temporal trial in the L- and T-compartment in the upper (A) and lower (B) panel, respectively. The colour codes for the R^2^-value and ‘.’ indicates a *p*-value between 0.05 and 0.1, ‘*’ indicates a *p*-value between 0.01 and 0.05, ‘**’ a *p*-value between 0.01 and 0.001. The *p*_adj_-values using Benjamini–Hochberg correction for multiple testing are not displayed as they were all non-significant. **Figure S7.** Comparison of the T-compartment of six tree individuals in the temporal trial. Trees 1 to 3 and trees 4 to 6 were standing adjacently in separate opposite rows. (A) Pairwise PERMANOVA with *p*-values adjusted using Benjamini–Hochberg correction for multiple testing. The colour codes for the R^2^-value and ‘.’ indicates a *p*_adj_-value between 0.05 and 0.1, ‘*’ indicates a *p*_adj_-value between 0.01 and 0.05. (B) Differentially abundant ASVs identified by ANCOM-BC. The heat map shows the coefficients obtained from the ANCOM-BC log-linear model divided by their standard error (called W-value). A “*” is shown if ANCOM-BC showed significant differences using the *p*_adj_-value in this comparison. The colour code indicates differential abundances between two samples with red indicating enrichment in the tree with the higher identifier number. The mean relative abundance of the ASVs in the T-compartment is shown as % and ASVs with mean abundances ≥0.3% are displayed. The ASVs in the rows of the heat map are separated according to phylum. **Figure S8.** Differentially abundant ASVs in the L- and T-compartment (upper and lower panel, respectively) for two different root size sections at four different time points according to ANCOM-BC. Fine roots had a diameter between 1 and 3 mm and thick roots between 3 and 6 mm. Samples were taken at four time points (TP1: 21.03.2019; TP2: 15.04.2019; TP3: 05.06.2019 and TP4: 20.08.2019). The first four columns compare the fine to the thick roots at each time point, the next three the different time points in the fine roots and the last three columns compare the thick roots at each time point. The heat map shows the coefficients obtained from the ANCOM-BC log-linear model divided by their standard error (called W-value). The colour code indicates differential abundances between two factors with red indicating enrichment in the second mentioned factor. A “*” is shown if ANCOM-BC showed significant differences using *p*_adj_-values in this comparison. The mean abundance of the ASVs in the entire compartment is shown as % and only ASVs with mean abundances ≥ 0.3% are shown. **Table S1.** The sampling time points of the temporal trial (left) and the spatio-temporal (ST) trial (right). **Table S2.** Sequences of bar coded forward primers targeting the 16SrRNA gene. Primers include a bar code (8 bp) and the primer sequence itself. The reverse primer was not modified. **Table S3.** The hierarchies in each of the trials with the number of samples. Total read number after quality filtering, mean number of reads per sample and the number of samples remaining after quality filtering. **Table S4.** The ten most prominent genera in each trial with their mean relative abundance and standard deviation (SD). **Table S5.** Differences in bacterial beta diversity independence on tree individual and root size section in the loosely (L) and tightly (T) associated root microbiota in the spatial trial. Effect sizes in beta diversity were assessed by pairwise PERMANOVA based on DEICODE distance matrices and *p*_adj_-values calculated using Bonferroni’s algorithm. **Table S6.** Significant differences in the apple root-associated bacterial community structure due to temporal, root size and spatial effects. The spatial effects in terms of tree-to-tree variation, longitudinal position of the tree and row of the tree were analysed separately. Effect sizes were analysed by PERMANOVA based on DEICODE distance matrices. Significant results are printed in bold.

## Data Availability

Sequence files and metadata for all samples used in this study have been deposited in Figshare (https://figshare.com/projects/Spatio-temporal_variation_in_the_root-associated_microbiota_of_orchard-grown_apple_trees/125116). A full record of all statistical analyses using the knitr package in R, metadata, the unrarefied ASV tables and the corresponding taxonomic classifications are available in GitHub (https://github.com/mfbeuq/becker_etal_spatiotemporal).

## Abbreviations

ASV: Amplicon sequence variant
CAP: Constrained analysis of principal coordinates
DLR: Dienstleistungszentrum Ländlicher Raum
FDR: False discovery rate
FR: Fine roots
L: Loosely associated
*p*_*adj*_: Adjusted *p*-values
T: Tightly associated
TR: Thick roots
