# Supplementary material for: Spatio-temporal variation in the root-associated microbiota of orchard-grown apple trees
Source: Environ Microbiome. 2022 Jun 17;17:31. doi: 10.1186/s40793-022-00427-z (PMC9205072; doi:10.1186/s40793-022-00427-z)
Supplement: Supplementary file 1 — Additional file 1. Figure S1. Photographs showing the root system of a fully grown commercial apple tree (top) and two rows of an apple orchard (bottom). Figure S2. Differential abundance analysis of the loosely (L) and tightly (T) associated bacteria in the three experimental field trials using ANCOM-BC. The heatmap shows the coefficients obtained from the ANCOM-BC log-linear model divided by their standard error (called W-value) with red indicating enrichment in the T-compartment. A is shown if ANCOM-BC showed significant differences using the padj-value in this comparison. The mean abundance of the families in their respective trial are shown in the adjacent barplot as % and only families with mean abundances ≥ 0.5% are shown (ST refers to the spatio-temporal trial). A greyed-out field means that this family is below the 0.5% threshold in a trial. The families in the heatmap rows are separated by the phylum they belong to and displayed in different colours. Figure S3. Root-associated bacterial community composition of the loosely (L) and tightly (T) associated bacteria in four different root size sections and the bulk soil (b) of four apple trees analysed in the spatial trial. Constrained analysis of principle coordinates (CAP; based on DEICODE distance matrix and the variables compartment, tree and root quadrant) to assess the relevance of those variables on variation in bacterial community composition. Figure S4. Root-associated bacterial community composition of bulk soil near the apple trees analysed in the spatial trial. Constrained analysis of principle coordinates (CAP; based on DEICODE distance matrix and the variables tree and root quadrant) to assess the relevance of those variables on variation in bacterial community composition. Figure S5. Differential abundance analysis of the loosely (L) and tightly (T) associated bacteria and the bulk soil (B) in four different trees (T1 to T4) of the spatial trial using ANCOM-BC. The heat map shows the coefficient [file 40793_2022_427_MOESM1_ESM.pdf]

Supplemental

Maximilian Becker, Manfred Hellmann, Claudia Knief

Spatio-temporal variation in the root-associated microbiota of orchard-grown apple trees

DNA extraction and 16S rRNA gene PCR

DNA extractions were performed using the NucleoSpin® Soil DNA extraction kit (Macherey Nagel, Düren, Germany). For the L-compartment and bulk soil samples 400 mg of dry soil were weighed into kit-supplied 2-ml MN Bead Tubes Type A and extraction was done according to the manufacturer´s instructions. For the T-samples two 2-ml MN Bead Tubes Type A were filled up to the 1-ml mark with grounded root material per sample and processed according to instructions until step 7 in the protocol (03/2019, Rev. 08 version). At step 7, the solution of two parallel tubes per sample were successively loaded onto the NucleoSpin® Soil Column and therewith pooled. The following steps were again performed according to manufacturer´s instructions with a final elution in 50 µl of PCR-grade water. The DNA concentrations were quantified using the QuantiFluor®dsDNA System (Promega Corporation, Fitchburg, WI) according to the manufacturer´s instructions and bulk soil or L-samples were subsequently diluted to 10 ng/µl, while T-samples were diluted to 30 ng/µl using PCR-grade water. For bacterial community analysis, the 16S rRNA gene was amplified using an LNA PCR protocol to suppress the amplification of plant organelle derived 16S rRNA genes [39]. The bacterial genes were amplified using the modified primer set 63f-1492r, followed by a nested PCR using primer set 799f-1193r (V5 - V7 region) to obtain PCR products of adequate length for sequencing. The first PCR was performed in triplicate assays per sample. Each 11-µl reaction contained 2 µl of 5x Herculase II reaction buffer (Agilent Technologies, Santa Clara, CA), 0.91 mM $MgCl_2$, 0.73 mg/ml BSA, 0.23 mM of dNTPs, 0.14 µM of each bacterial primer (BioTez, Berlin, Germany), 0.55 µM of each LNA primer (Qiagen, Hilden, Germany), 0.5 U of Herculase II DNA polymerase and 1 µl of DNA template. Thermal cycling conditions were: an initial denaturation at 95 °C for 2 min followed by 25 cycles of 95 °C for 20 sec, 70 °C for 20 sec (LNA primer annealing), 56 °C for 20 sec (bacterial 16S rRNA gene primer annealing), 72 °C for 45 sec and a final elongation step at 72 °C for 3 min. The obtained triplicate PCR products per sample were pooled, 10-fold diluted with PCR grade water and used as template in the second, nested PCR for sample-specific barcoding. Each 30-µl nested PCR assay contained 6 µl of 5x Herculase II reaction buffer, 1 mM $MgCl_2$, 0.6 mg/ml BSA, 0.25 mM dNTPs, 0.25 µM of each primer, 1.5 U of Herculase II DNA polymerase and 3 µl template DNA. The forward primer in this nested PCR contained an 8-bp sample-specific barcode (table S2), similarly as used in Frindte et al. [40]. Thermal cycling conditions were: an initial denaturation at 95 °C for 1 min followed by 10 cycles of 95 °C for 20 sec, 56 °C for 20 sec, 72 °C for 30 sec and a final elongation step at 72 °C for 2:30 min. Successful amplification was validated by agarose gel-electrophoresis. PCR products were quantified using the QuantiFluor dsDNA System on an Infinite 200 Pro plate reader (Tecan, Männedorf, Switzerland) at 490 nm excitation and 530 nm emission wavelength. Afterwards, PCR products were pooled at equimolar concentrations and purified with the HighPrep™PCR Clean-up System kit (MagBio Genomics, Gaithersburg, MD). Library preparation and sequencing on a HiSeq system (Illumina, San Diego, CA) was performed by the Max Planck-Genome-centre Cologne and generated paired-end reads (2 × 250 bp).

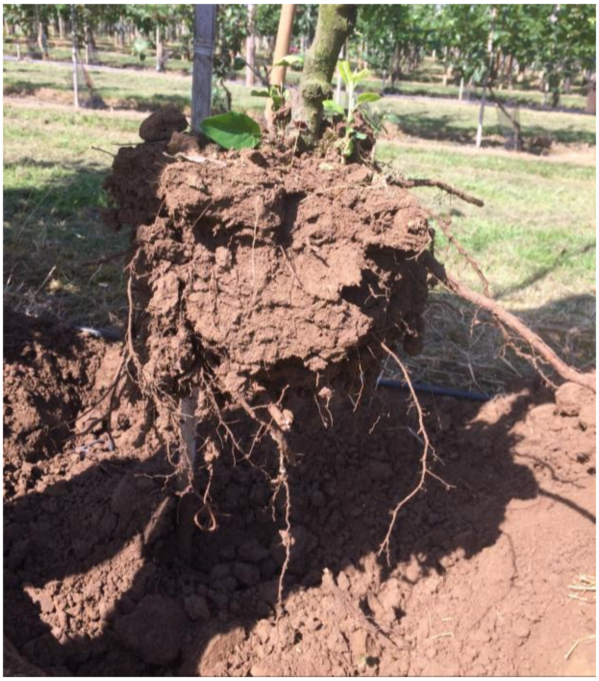

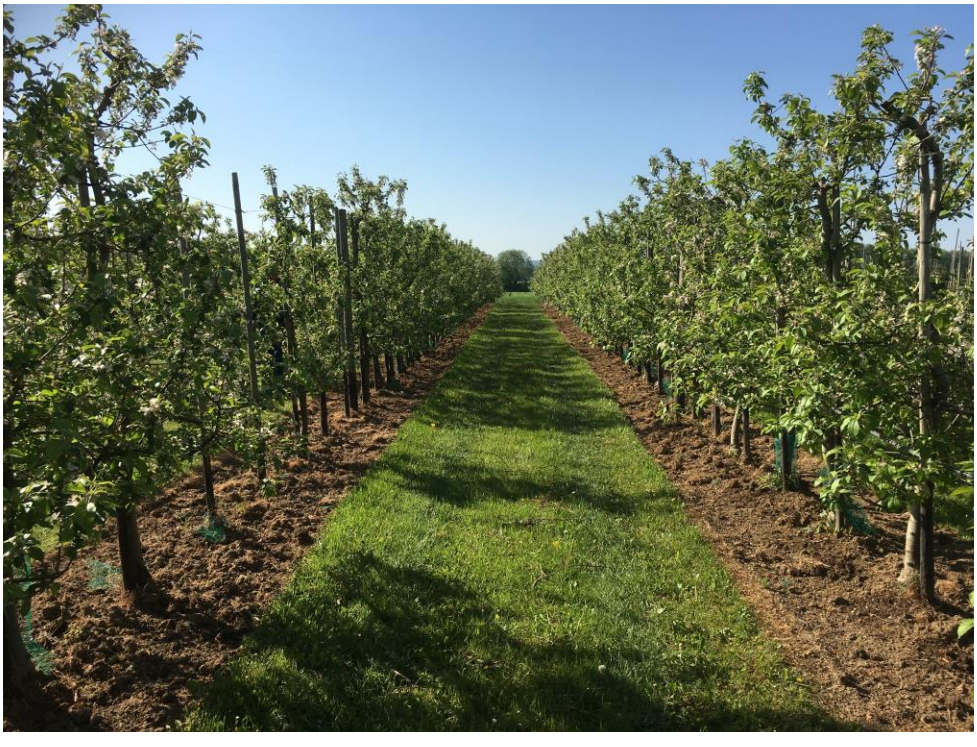

Figure S1. Photographs showing the root system of a fully grown commercial apple tree (top) and two rows of an apple orchard (bottom).

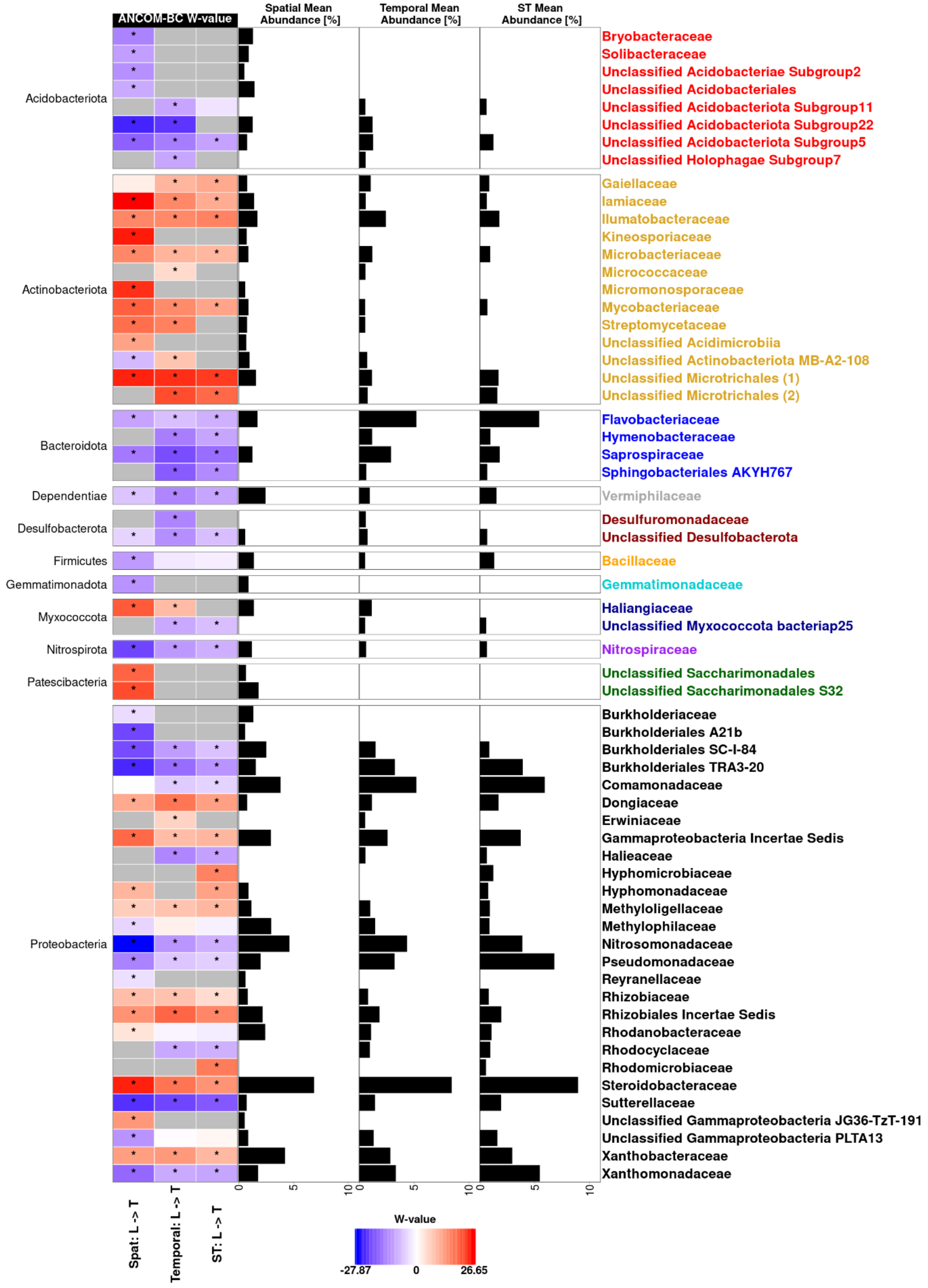

Figure S2. Differential abundance analysis of the loosely (L) and tightly (T) associated bacteria in the three experimental field trials using ANCOM-BC. The heatmap shows the coefficients obtained from the ANCOM-BC log-linear model divided by their standard error (called W-value) with red indicating enrichment in the T-compartment. A "*" is shown if ANCOM-BC showed significant differences using the p_{adj}-value in this comparison. The mean abundance of the families in their respective trial are shown in the adjacent barplot as % and only families with mean abundances ≥ 0.5 % are shown (ST refers to the spatio-temporal trial). A greyed-out field means that this family is below the 0.5 % threshold in a trial. The families in the heatmap rows are separated by the phylum they belong to and displayed in different colours.

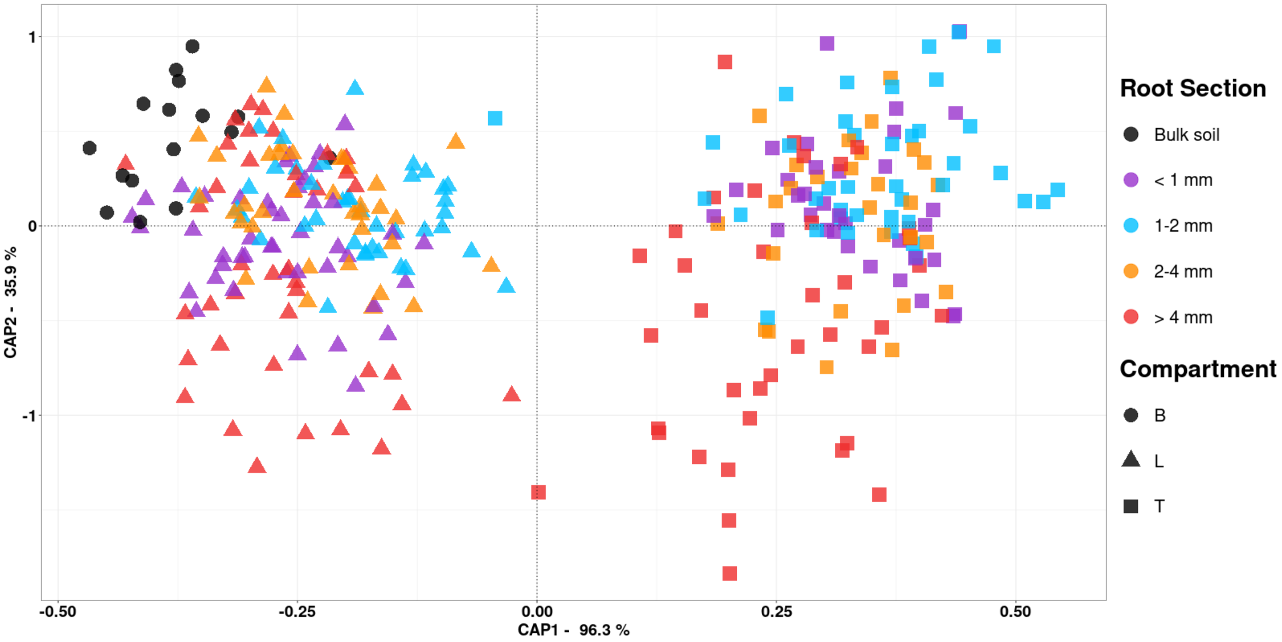

Figure S3. Root-associated bacterial community composition of the loosely (L) and tightly (T) associated bacteria in four different root size sections and the bulk soil (b) of four apple trees analysed in the spatial trial. Constrained analysis of principle coordinates (CAP; based on DEICODE distance matrices and the variables compartment, tree and root quadrant) to assess the relevance of those variables on variation in bacterial community composition.

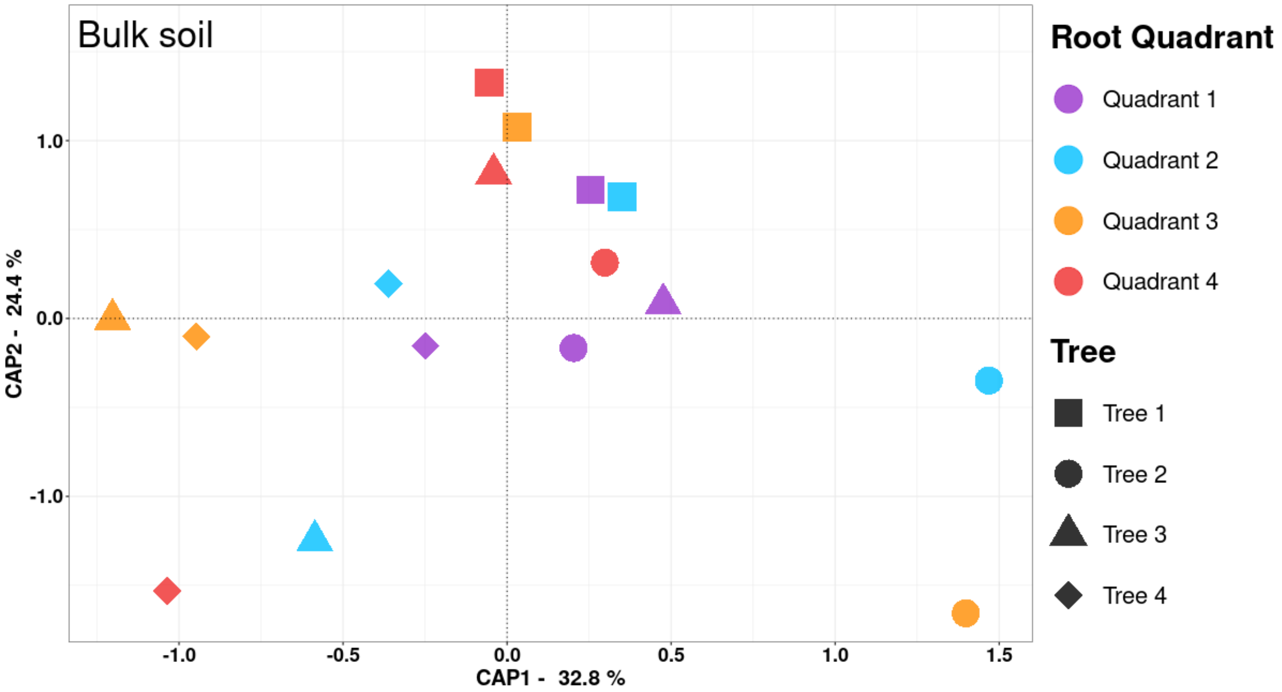

Figure S4. Root-associated bacterial community composition of bulk soil near the apple trees analysed in the spatial trial. Constrained analysis of principle coordinates (CAP; based on DEICODE distance matrices and the variables tree and root quadrant) to assess the relevance of those variables on variation in bacterial community composition.

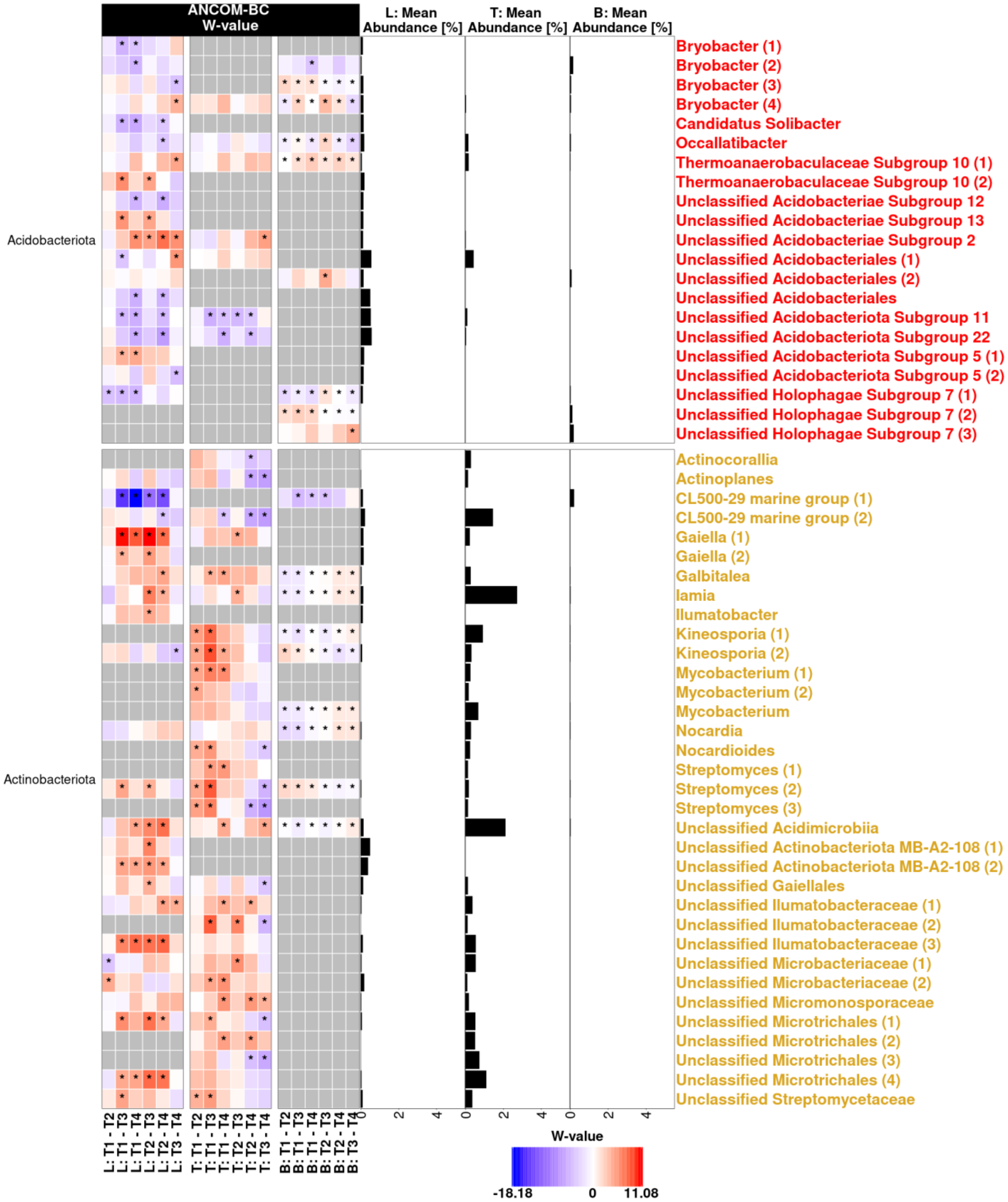

Figure S5 Part 1/4

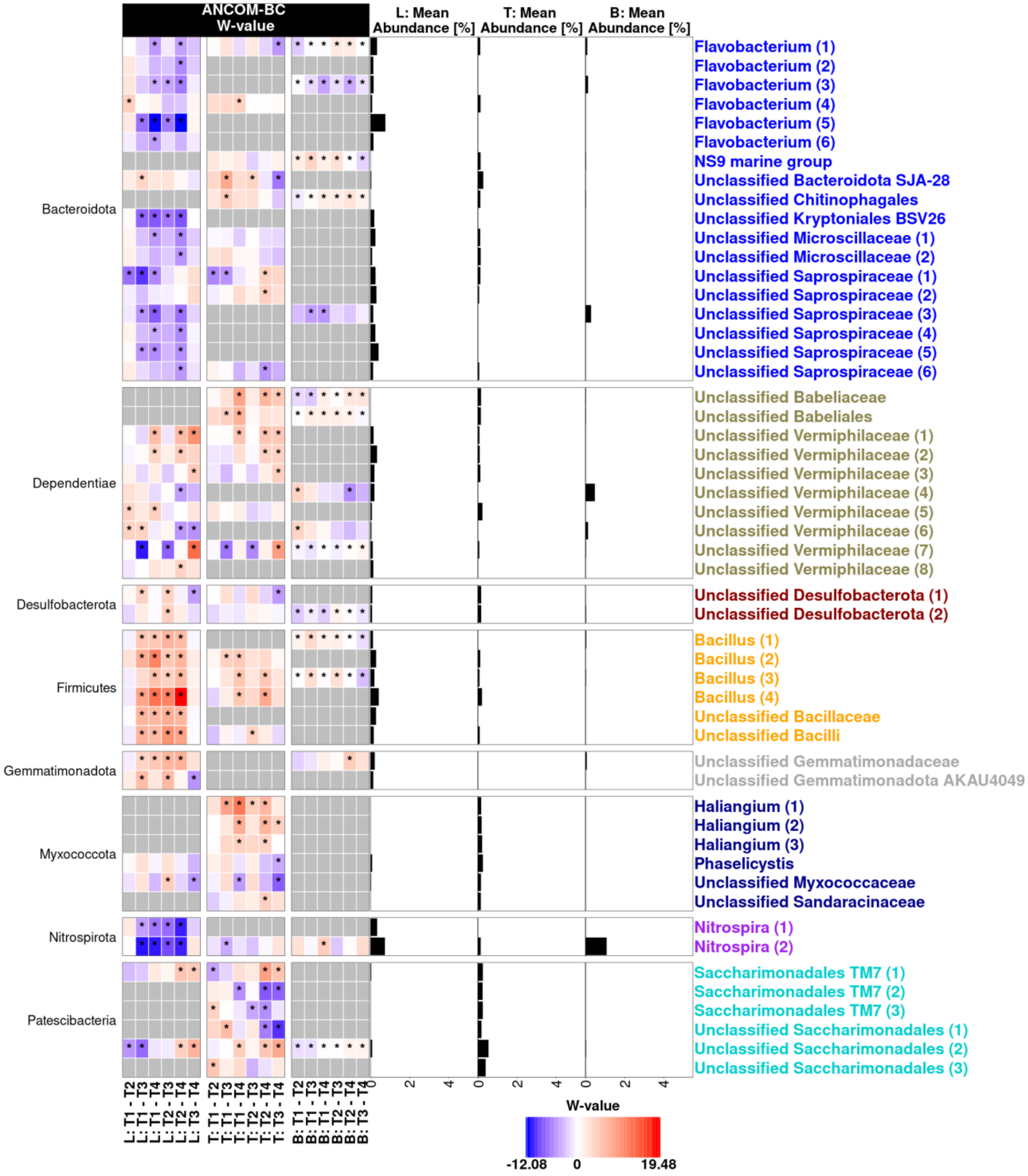

Figure S5 Part 2/4

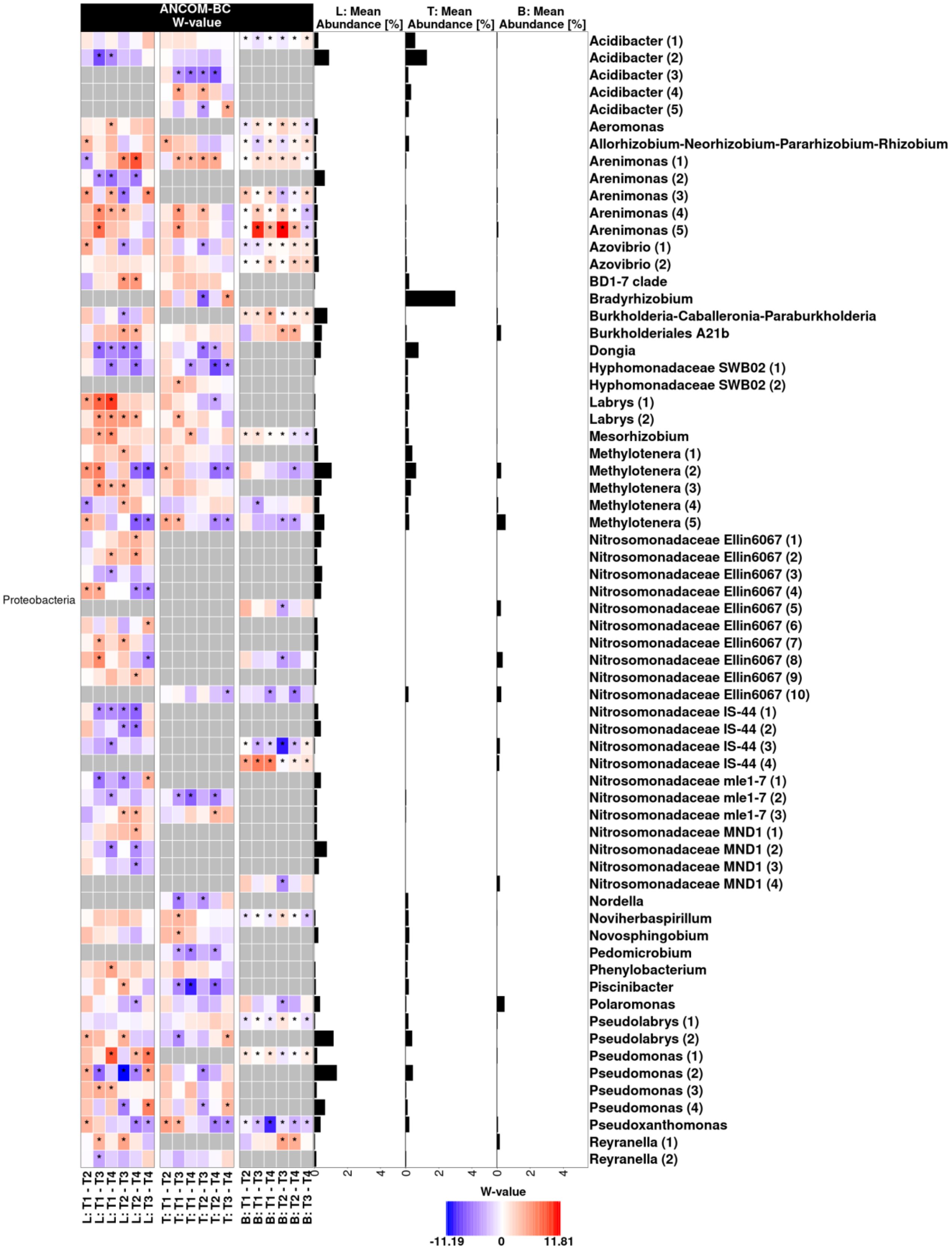

Figure S5 Part 3/4

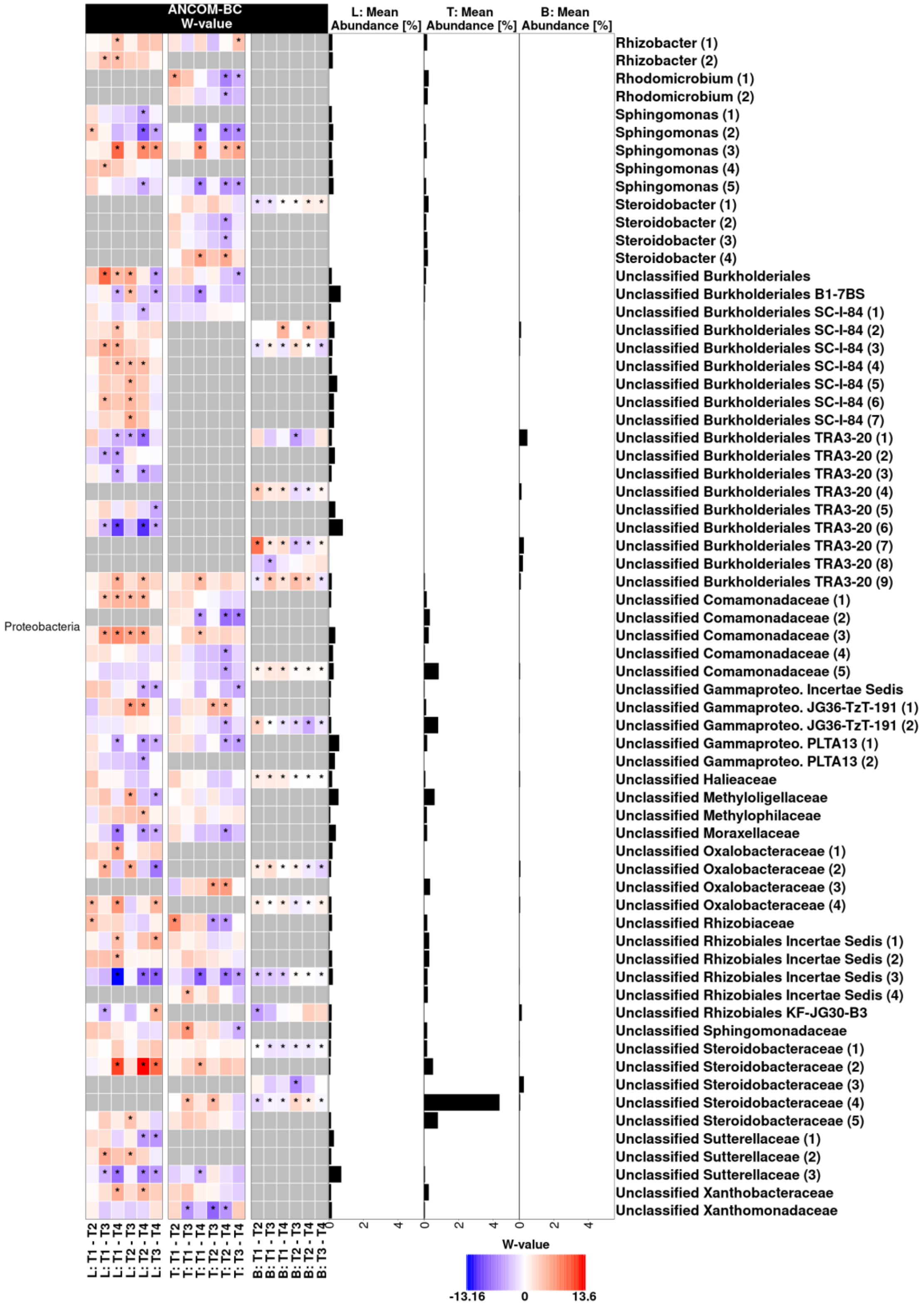

Figure S5 Part 4/4. Differential abundance analysis of the loosely (L) and tightly (T) associated bacteria and the bulk soil (b) in four different trees (T1 to T4) of the spatial trial using ANCOM-BC. The heatmap shows the coefficients obtained from the ANCOM-BC log-linear model divided by their standard error (called W-value). A "*" is shown if ANCOM-BC showed significant differences using the p_{adj}-value in this comparison. The colour code indicates differential abundances between two samples with red indicating enrichment in the larger root sections. A grey colour indicates that this ASV was not detected in the respective compartment. The mean relative abundance of the ASVs in the entire compartment is shown as % and ASVs with mean abundances ≥0.1 % in either compartment are displayed. The ASVs in the rows of the heatmap are separated according to phylum.

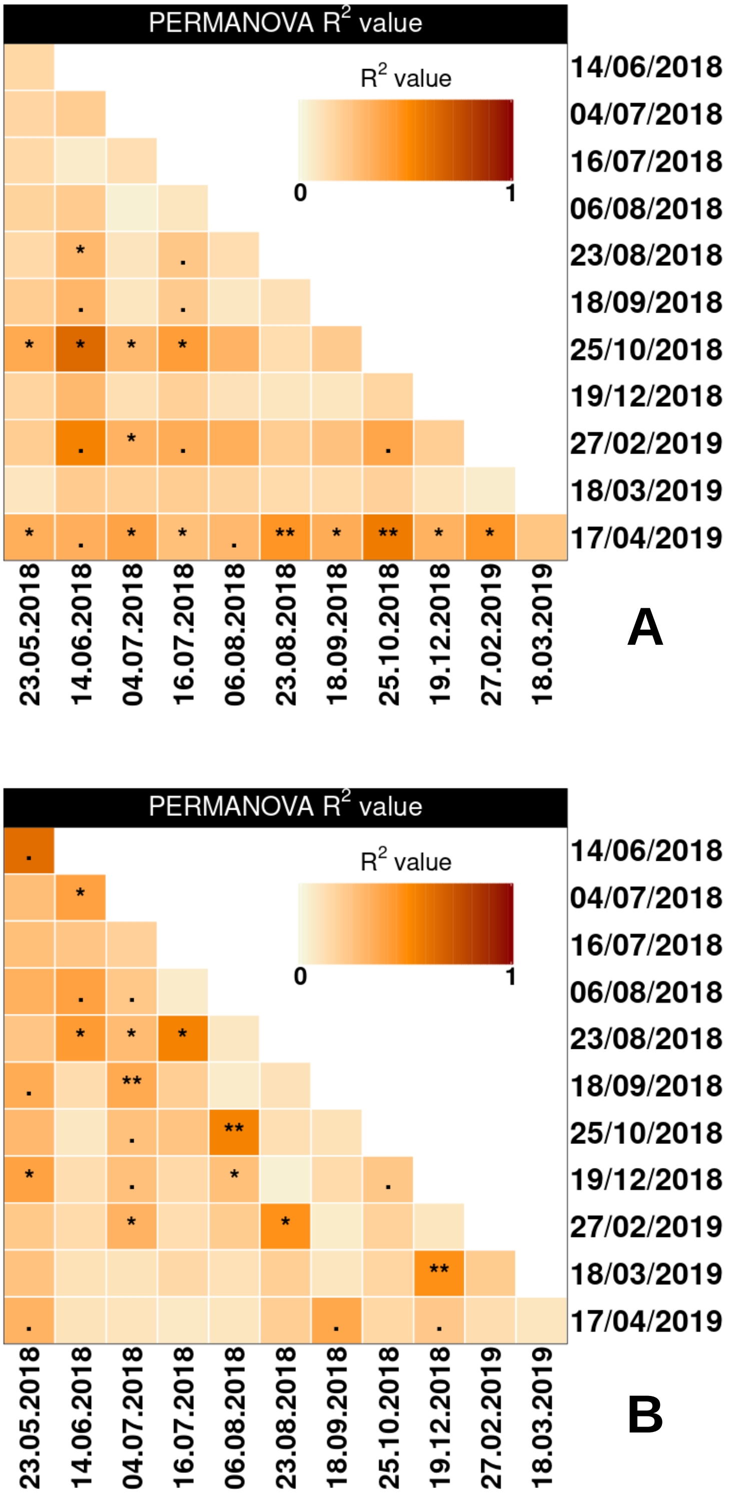

Figure S6. Pairwise PERMANOVA for comparison of timepoints in the temporal trial in the L- and T-compartment in the upper (A) and lower (B) panel, respectively. The colour codes for the R^2-value and `.` indicates a *p*-value between 0.05 and 0.1, `*` indicates a *p*-value between 0.01 and 0.05, `**` a *p*-value between 0.01 and 0.001. The p_{adj}-values using Benjamini-Hochberg correction for multiple testing are not displayed as they were all non-significant.

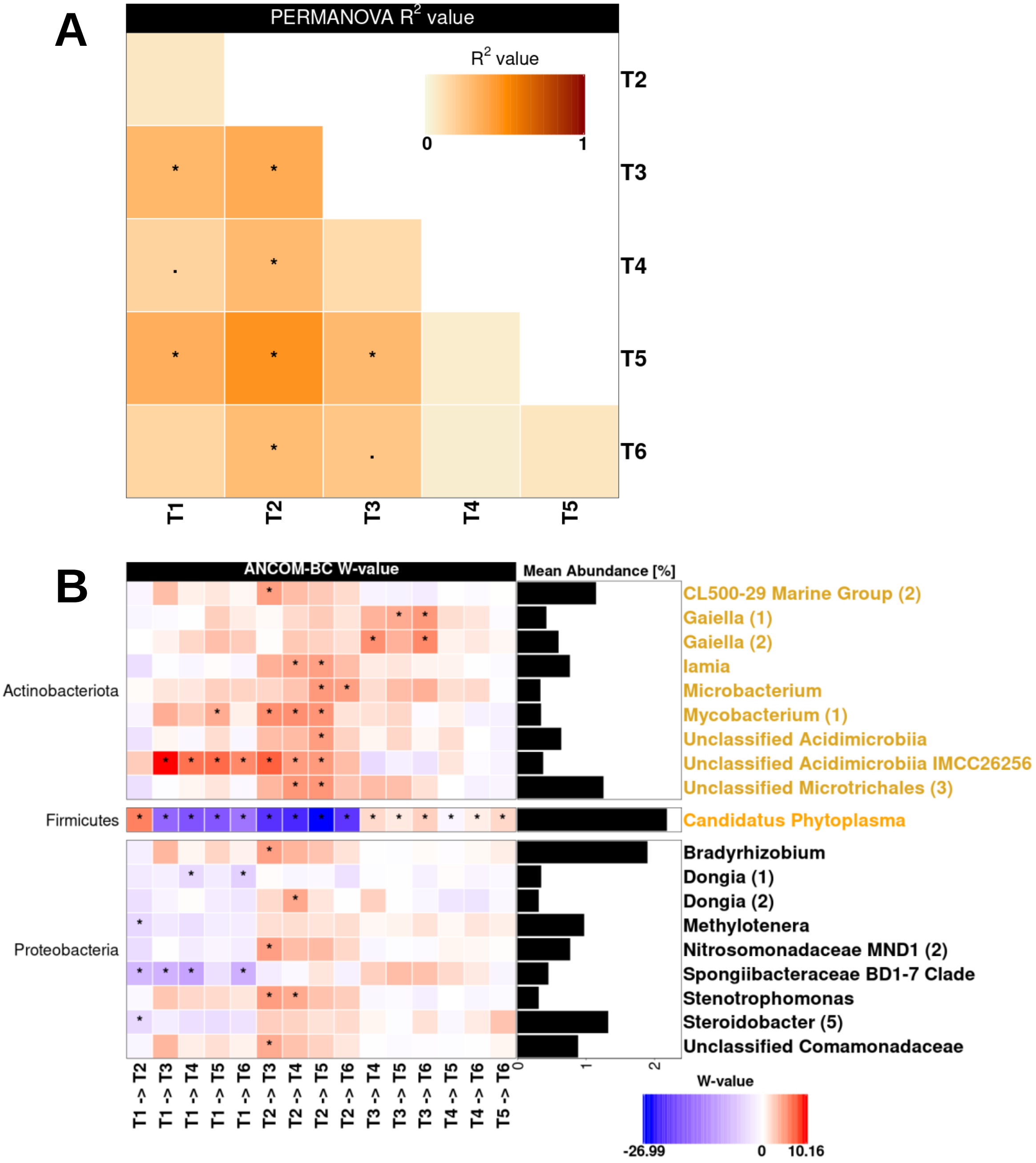

Figure S7. Comparison of the T-compartment of six tree individuals in the temporal trial. Trees 1 to 3 and trees 4 to 6 were standing adjacently in separate opposite rows. (A) Pairwise PERMANOVA with *p*-values adjusted using Benjamini-Hochberg correction for multiple testing. The colour codes for the R^2-value and `.` indicates a p_{adj}-value between 0.05 and 0.1, `*` indicates a p_{adj}-value between 0.01 and 0.05. (B) Differentially abundant ASVs identified by ANCOM-BC. The heatmap shows the coefficients obtained from the ANCOM-BC log-linear model divided by their standard error (called W-value). A "*" is shown if ANCOM-BC showed significant differences using the p_{adj}-value in this comparison. The colour code indicates differential abundances between two samples with red indicating enrichment in the tree with the higher identifier number. The mean relative abundance of the ASVs in the T- compartment is shown as % and ASVs with mean abundances ≥ 0.3 % are displayed. The ASVs in the rows of the heatmap are separated according to phylum.

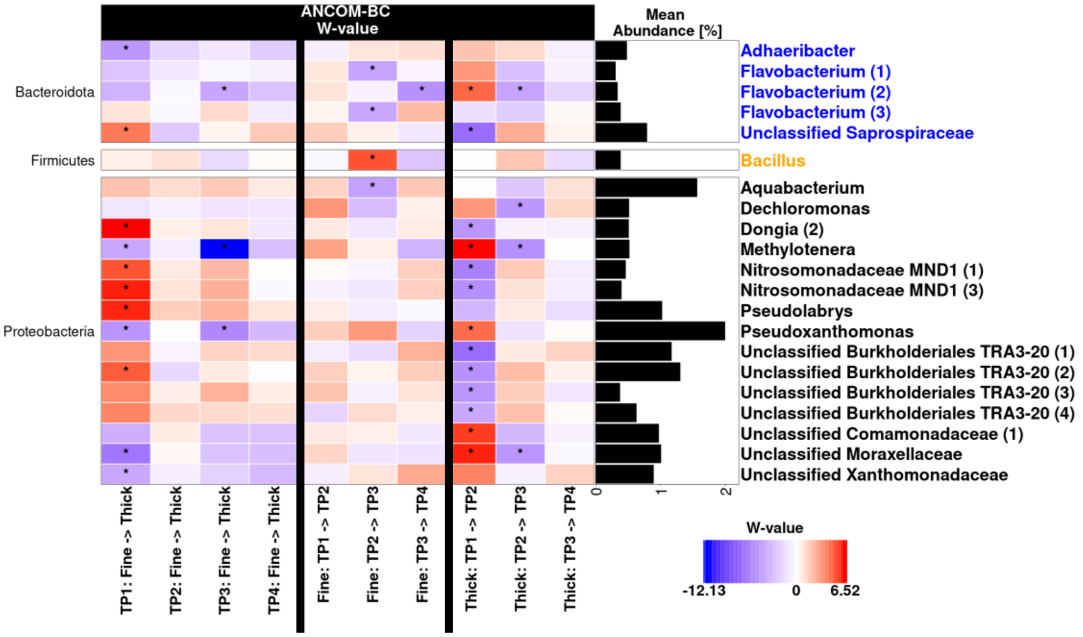

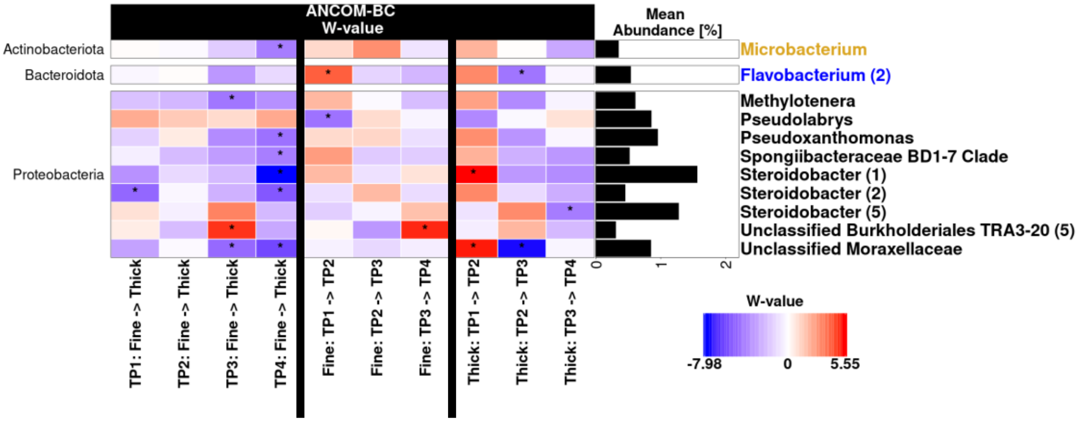

Figure S8. Differentially abundant ASVs in the L- and T-compartment (upper and lower panel, respectively) for two different root size sections at four different timepoints according to ANCOM-BC. Fine roots had a diameter between 1 and 3 mm and thick roots between 3 and 6 mm. Samples were taken at four timepoints (TP1: 21.03.2019; TP2: 15.04.2019; TP3: 05.06.2019 and TP4: 20.08.2019). The first four columns compare the fine to the thick roots at each timepoint, the next three the different timepoints in the fine roots and the last three columns compare the thick roots at each timepoint. The heatmap shows the coefficients obtained from the ANCOM-BC log-linear model divided by their standard error (called W-value). The colour code indicates differential abundances between two factors with red indicating enrichment in the second mentioned factor. A "*" is shown if ANCOM-BC showed significant differences using p_{adj}-values in this comparison. The mean abundance of the ASVs in the entire compartment is shown as % and only ASVs with mean abundances ≥ 0.3 % are shown.

Table S1. The sampling timepoints of the temporal trial (left) and the spatio-temporal (ST) trial (right).

Temporal trial		
**Timepoint 1**	**23.05.2018**	**Spring**
**Timepoint 2**	**14.06.2018**	**Summer**
**Timepoint 3**	**04.07.2018**	
**Timepoint 4**	**16.07.2018**	
**Timepoint 5**	**06.08.2018**	
**Timepoint 6**	**23.08.2018**	
**Timepoint 7**	**18.09.2018**	**Autumn**
**Timepoint 8**	**25.10.2018**	
**Timepoint 9**	**19.12.2018**	**Winter**
**Timepoint 10**	**27.02.2019**	
**Timepoint 11**	**18.03.2019**	
**Timepoint 12**	**17.04.2019**	**Spring**

ST trial		
**Timepoint 1**	**21.03.2019**	**Spring**
**Timepoint 2**	**15.04.2019**	
**Timepoint 3**	**05.06.2019**	**Summer**
**Timepoint 4**	**20.08.2019**	

Table S2: Sequences of barcoded forward primers targeting the 16S rRNA gene. Primers include a barcode (8 bp) and the primer sequence itself. The reverse primer was not modified.

Name	Barcode + Primer 799f
799f-BC1	AACACCTA AAC MGG ATT AGA TAC CCK G
799f-BC2	ACGTAGCT AAC MGG ATT AGA TAC CCK G
799f-BC3	ATATAGGA AAC MGG ATT AGA TAC CCK G
799f-BC4	CACAGTTG AAC MGG ATT AGA TAC CCK G
799f-BC5	CCTACAAC AAC MGG ATT AGA TAC CCK G
799f-BC6	CGTCGGCT AAC MGG ATT AGA TAC CCK G
799f-BC7	GACGTCAA AAC MGG ATT AGA TAC CCK G
799f-BC8	GCGTTTCG AAC MGG ATT AGA TAC CCK G
799f-BC9	GGTCTGAC AAC MGG ATT AGA TAC CCK G
799f-BC10	GTTTCACT AAC MGG ATT AGA TAC CCK G
799f-BC11	TCCAGCCT AAC MGG ATT AGA TAC CCK G
799f-BC12	TGCGGTTA AAC MGG ATT AGA TAC CCK G
799f-BC13	GCAGCCTC AAC MGG ATT AGA TAC CCK G
799f-BC14	GGCGAGGA AAC MGG ATT AGA TAC CCK G
799f-BC15	GTGGGATA AAC MGG ATT AGA TAC CCK G
799f-BC16	TATCTCCG AAC MGG ATT AGA TAC CCK G
799f-BC17	ACTAACTG AAC MGG ATT AGA TAC CCK G
799f-BC18	ATCCTATT AAC MGG ATT AGA TAC CCK G
799f-BC19	CACGTGTT AAC MGG ATT AGA TAC CCK G
799f-BC20	CCTTTACA AAC MGG ATT AGA TAC CCK G
799f-BC21	CTAGATTC AAC MGG ATT AGA TAC CCK G
799f-BC22	GAGAACTC AAC MGG ATT AGA TAC CCK G
799f-BC23	GCTCAGTT AAC MGG ATT AGA TAC CCK G
799f-BC24	GTACTTGC AAC MGG ATT AGA TAC CCK G
799f-BC25	TACGAATC AAC MGG ATT AGA TAC CCK G
799f-BC26	TCCTACTA AAC MGG ATT AGA TAC CCK G
799f-BC27	TGGTCTTC AAC MGG ATT AGA TAC CCK G
799f-BC28	AACCGTGT AAC MGG ATT AGA TAC CCK G
799f-BC29	GGTCCTTG AAC MGG ATT AGA TAC CCK G
799f-BC30	GTTGTCCC AAC MGG ATT AGA TAC CCK G
799f-BC31	TCATTAGG AAC MGG ATT AGA TAC CCK G
799f-BC32	TGATCCGA AAC MGG ATT AGA TAC CCK G
799f-BC33	ATCGCCAG AAC MGG ATT AGA TAC CCK G
799f-BC34	CAGGAGGC AAC MGG ATT AGA TAC CCK G
799f-BC35	CGAACTGT AAC MGG ATT AGA TAC CCK G
799f-BC36	CTAGTCAT AAC MGG ATT AGA TAC CCK G
799f-BC37	GAGTTAAC AAC MGG ATT AGA TAC CCK G
799f-BC38	GCTGGCGA AAC MGG ATT AGA TAC CCK G
799f-BC39	GTAGAGCT AAC MGG ATT AGA TAC CCK G
799f-BC40	TACTGCGC AAC MGG ATT AGA TAC CCK G
799f-BC41	TCGCGTAC AAC MGG ATT AGA TAC CCK G
799f-BC42	TGTAGGTC AAC MGG ATT AGA TAC CCK G
799f-BC43	AAGCGGTC AAC MGG ATT AGA TAC CCK G
799f-BC44	ACTCTAAG AAC MGG ATT AGA TAC CCK G
799f-BC45	TGAGAGTG AAC MGG ATT AGA TAC CCK G
799f-BC46	TTCTGATG AAC MGG ATT AGA TAC CCK G
799f-BC47	ACAGTGCA AAC MGG ATT AGA TAC CCK G
799f-BC48	AGTAGTGG AAC MGG ATT AGA TAC CCK G

Table S3. The hierarchies in each of the trials with the number of samples. Total read number after quality filtering, mean number of reads per sample and the number of samples remaining after quality filtering.

Trial	Hierarchical structure	Number of samples	Total number of reads	Mean number of reads / sample	Samples remaining after quality filtering
Spatial trial	4 trees x 4 quadrants x 4 size classes x 3 pseudo-replications x 2 compartments	384 root associated samples	11.494.965	36.725	297 samples
	4 trees x 4 quadrants	16 bulk soil samples			16 samples
Temporal trial	6 trees x 12 time points x 2 compartments	144 root associated samples	4.248.425	37.932	112 samples
Combined trial	9 trees x 2 size classes x 4 time points x 2 compartments	144 root associated samples	3.650.109	35.785	102 samples

Table S4. The ten most prominent genera in each trial with their mean relative abundance and standard deviation (SD).

		Class	Order	Family	Genus	Mean	SD
**SPATIAL**	**L**	*Gammaproteobacteria*	*Burkholderiales*	SC-I-84		4.2	1.5
		Gammaproteobacteria	*Burkholderiales*	*Nitrosomonadaceae*	Ellin6067	3.8	1.5
		Babeliae	*Babeliales*	*Vermiphilaceae*		3.1	1.4
		Gammaproteobacteria	*Pseudomonadales*	*Pseudomonadaceae*	*Pseudomonas*	3.0	2.1
		Gammaproteobacteria	*Burkholderiales*	TRA3-20		2.8	1.1
		Gammaproteobacteria	*Burkholderiales*	*Methylophilaceae*	*Methylotenera*	2.7	1.6
		Bacteroidia	*Flavobacteriales*	*Flavobacteriaceae*	*Flavobacterium*	2.5	1.5
		Acidobacteriota - Subgroup22				2.2	0.9
		Gammaproteobacteria	*Burkholderiales*	*Comamonadaceae*		2.2	1.0
		Gammaproteobacteria	*Steroidobacterales*	*Steroidobacteraceae*		2.1	1.0
	**T**	*Gammaproteobacteria*	*Steroidobacterales*	*Steroidobacteraceae*		10.9	3.3
		Gammaproteobacteria	Incertae Sedis	Unknown Family	*Acidibacter*	4.4	1.1
		Alphaproteobacteria	*Rhizobiales*	*Xanthobacteraceae*	*Bradyrhizobium*	3.6	0.9
		Saccharimonadia	*Saccharimonadales*	S32	TM7	3.3	1.8
		Acidimicrobiia				3.3	1.3
		Acidimicrobiia	*Microtrichales*			3.0	1.5
		Acidimicrobiia	*Microtrichales*	*Iamiaceae*	*Iamia*	2.8	1.6
		Alphaproteobacteria	*Rhizobiales*	Incertae Sedis		2.6	1.2
		Polyangia	*Haliangiales*	*Haliangiaceae*	*Haliangium*	2.3	0.8
		Gammaproteobacteria	*Burkholderiales*	*Comamonadaceae*		2.1	1.0
**TEMPORAL**	**L**	*Bacteroidia*	*Flavobacteriales*	*Flavobacteriaceae*	*Flavobacterium*	6.6	3.7
		Gammaproteobacteria	*Burkholderiales*	TRA3-20		5.1	1.6
		Bacteroidia	*Chitinophagales*	*Saprospiraceae*		4.8	1.4
		Gammaproteobacteria	*Pseudomonadales*	*Pseudomonadaceae*	*Pseudomonas*	4.1	3.9
		Gammaproteobacteria	*Burkholderiales*	*Comamonadaceae*		2.8	0.9
		Gammaproteobacteria	*Burkholderiales*	*Nitrosomonadaceae*	Ellin6067	2.8	0.6
		Gammaproteobacteria	*Burkholderiales*	*Sutterellaceae*		2.4	0.5
		Gammaproteobacteria	*Burkholderiales*	*Nitrosomonadaceae*	MND1	2.4	0.8
		Gammaproteobacteria	*Burkholderiales*	SC-I-84		2.2	0.6
		Acidobacteriota - Subgroup22				2.1	0.6
	**T**	*Gammaproteobacteria*	*Steroidobacterales*	*Steroidobacteraceae*	*Steroidobacter*	12.3	4.5
		Bacteroidia	*Flavobacteriales*	*Flavobacteriaceae*	*Flavobacterium*	3.7	4.2
		Gammaproteobacteria	Incertae Sedis	Unknown Family	*Acidibacter*	3.3	1.1
		Acidimicrobiia	*Microtrichales*	*Ilumatobacteraceae*	CL500-29 Marine Group	2.6	1.4
		Alphaproteobacteria	*Rhizobiales*	Incertae Sedis		2.5	1.1
		Gammaproteobacteria	*Burkholderiales*	*Comamonadaceae*		2.4	0.7
		Gammaproteobacteria	*Pseudomonadales*	*Pseudomonadaceae*	*Pseudomonas*	2.2	2.6
		Bacilli	*Acholeplasmatales*	*Acholeplasmataceae*	Candidatus *Phytoplasma*	2.2	5.1
		Acidimicrobiia	*Microtrichales*			2.2	0.9
		Alphaproteobacteria	*Rhizobiales*	*Xanthobacteraceae*	*Bradyrhizobium*	2.1	0.7
**FIELD**	**L**	*Gammaproteobacteria*	*Pseudomonadales*	*Pseudomonadaceae*	*Pseudomonas*	8.7	5.4
		Bacteroidia	*Flavobacteriales*	*Flavobacteriaceae*	*Flavobacterium*	7.3	4.2
		Gammaproteobacteria	*Burkholderiales*	TRA3-20		5.6	2.5
		Gammaproteobacteria	*Burkholderiales*	*Comamonadaceae*		3.8	2.1
		Gammaproteobacteria	*Burkholderiales*	*Sutterellaceae*		3.0	1.2
		Bacteroidia	*Chitinophagales*	*Saprospiraceae*		2.8	1.0
		Gammaproteobacteria	*Burkholderiales*	*Nitrosomonadaceae*	MND1	2.6	1.4
		Gammaproteobacteria	*Xanthomonadales*	*Xanthomonadaceae*	*Pseudoxanthomonas*	2.5	3.1
		Gammaproteobacteria	*Steroidobacterales*	*Steroidobacteraceae*		2.4	0.7
		Babeliae	*Babeliales*	*Vermiphilaceae*		2.3	1.5
	**T**	*Gammaproteobacteria*	*Steroidobacterales*	*Steroidobacteraceae*	*Steroidobacter*	15.7	4.7
		Gammaproteobacteria	Incertae Sedis	Unknown Family	*Acidibacter*	6.1	1.9
		Gammaproteobacteria	*Pseudomonadales*	*Pseudomonadaceae*	*Pseudomonas*	4.7	5.6
		Acidimicrobiia	*Microtrichales*			4.0	1.9
		Bacteroidia	*Flavobacteriales*	*Flavobacteriaceae*	*Flavobacterium*	3.1	3.6
		Alphaproteobacteria	*Dongiales*	*Dongiaceae*	*Dongia*	3.1	1.0
		Alphaproteobacteria	*Rhizobiales*	Incertae Sedis		2.8	0.8
		Acidimicrobiia	*Microtrichales*	*Ilumatobacteraceae*		2.4	1.1
		Alphaproteobacteria	*Rhizobiales*	*Hyphomicrobiaceae*	*Pedomicrobium*	2.4	1.0
		Alphaproteobacteria	*Rhizobiales*	*Xanthobacteraceae*	*Bradyrhizobium*	2.0	0.7

Table S5. Differences in bacterial beta diversity in dependence on tree individual and root size section in the loosely (L) and tightly (T) associated root microbiota in the spatial trial. Effect sizes in beta diversity were assessed by pairwise PERMANOVA based on DEICODE distance matrices and p_{adj}-values calculated using Bonferroni's algorithm.

Compartment	Pairs	F.Model	R^2	*p*-value	p_{adj}-value
L	T1 vs T2	1.736	0.023	0.179	1.000
	T1 vs T3	37.763	0.332	0.001	0.006
	T1 vs T4	46.009	0.365	0.001	0.006
	T2 vs T3	26.828	0.266	0.001	0.006
	T2 vs T4	36.274	0.317	0.001	0.006
	T3 vs T4	6.472	0.075	0.001	0.006
	< 1 mm vs 1-2 mm	6.661	0.076	0.002	0.012
	< 1 mm vs 2-4 mm	16.862	0.178	0.001	0.006
	< 1 mm vs > 4 mm	17.485	0.178	0.001	0.006
	1-2 mm vs 2-4 mm	3.281	0.043	0.026	0.156
	1-2 mm vs > 4 mm	9.258	0.109	0.001	0.006
	2-4 mm vs > 4 mm	4.636	0.060	0.003	0.018
T	T1 vs T2	0.393	0.006	0.803	1.000
	T1 vs T3	4.321	0.060	0.008	0.048
	T1 vs T4	7.323	0.093	0.001	0.006
	T2 vs T3	3.384	0.050	0.021	0.126
	T2 vs T4	7.638	0.102	0.001	0.006
	T3 vs T4	4.986	0.066	0.002	0.012
	< 1 mm vs 1-2 mm	10.758	0.132	0.001	0.006
	< 1 mm vs 2-4 mm	23.152	0.282	0.001	0.006
	< 1 mm vs > 4 mm	32.237	0.318	0.001	0.006
	1-2 mm vs 2-4 mm	6.994	0.096	0.002	0.012
	1-2 mm vs > 4 mm	29.427	0.279	0.001	0.006
	2-4 mm vs > 4 mm	12.437	0.163	0.001	0.006

Table S6. Significant differences in the apple root-associated bacterial community structure due to temporal, root size and spatial effects. The spatial effects in terms of tree-to-tree variation, longitudinal position of the tree and row of the tree were analysed separately. Effect sizes were analysed by PERMANOVA based on DEICODE distance matrices. Significant results are printed in bold.

Compart-ment	Input Variable	Variable	PERMANOVA		
			F.Model	R^2	*p*
L	Tree	**Timepoint**	**2.978**	**0.090**	**0.006**
		**Root section**	**13.681**	**0.137**	**0.001**
		**Tree**	**3.637**	**0.292**	**0.001**
		Timepoint * Root section	1.935	0.058	0.076
	Row	**Timepoint**	**2.215**	**0.090**	**0.017**
		**Root section**	**10.175**	**0.137**	**0.001**
		**Row**	**5.971**	**0.081**	**0.016**
		Timepoint * Root section	1.384	0.056	0.139
		Timepoint * Row	0.725	0.029	0.572
		Root section * Row	0.583	0.008	0.532
		Timepoint * Root section * Row	0.770	0.031	0.508
	Longitudinal position	**Timepoint**	**2.617**	**0.090**	**0.014**
		**Root section**	**12.022**	**0.137**	**0.001**
		**Longitudinal position**	**5.652**	**0.129**	**0.014**
		Timepoint * Root section	1.738	0.060	0.089
		Timepoint * Longitudinal position	0.760	0.052	0.619
		Root section * Longitudinal position	0.504	0.012	0.691
		**Timepoint * Root section *Longitudinal position**	**1.916**	**0.131**	**0.025**
T	Tree	**Timepoint**	**2.980**	**0.119**	**0.018**
		**Root section**	**7.259**	**0.096**	**0.001**
		**Tree**	**3.248**	**0.345**	**0.002**
		Timepoint * Root section	1.680	0.067	0.112
	Row	**Timepoint**	**2.010**	**0.119**	**0.043**
		**Root section**	**4.896**	**0.096**	**0.006**
		**Row**	**3.970**	**0.078**	**0.010**
		Timepoint * Root section	0.972	0.057	0.379
		Timepoint * Row	0.741	0.044	0.564
		Root section * Row	0.768	0.015	0.410
		Timepoint * Root section *Row	0.646	0.038	0.605
	Longitudinal position	Timepoint	1.739	0.119	0.068
		**Root section**	**4.236**	**0.096**	**0.011**
		Longitudinal position	2.040	0.093	0.308
		Timepoint * Root section	1.128	0.077	0.309
		Timepoint * Longitudinal position	0.426	0.058	0.928
		Root section * Longitudinal position	0.234	0.011	0.908
		Timepoint * Root section *Longitudinal position	0.489	0.045	0.777
